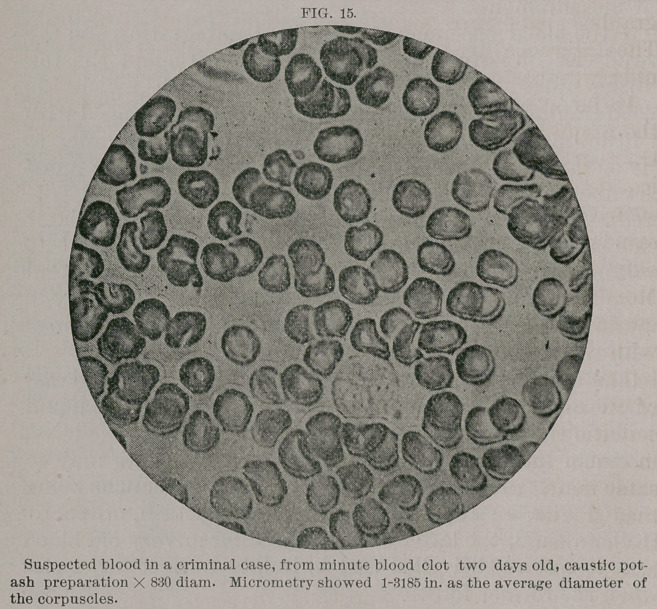# Comparative Studies of Mammalian Blood

**Published:** 1888-07

**Authors:** Henry F. Formad

**Affiliations:** Lecturer on Experimental Pathology and Demonstrator of Morbid Anatomy, in the University of Pennsylvania—Coroner's Physician of Philadelphia, etc.


### ART. XIX.—COMPARATIVE STUDIES OF MAMMALIAN BLOOD.

### WITH SPECIAL REFERENCE TO THE MICROSCOPICAL DIAGNOSIS OF BLOOD STAINS IN CRIMINAL CASES.*

BY HENRY F. FORMAD, B.M.,M.D.,

*Lecturer on Experimental Pathology and Demonstrator of Morbid Anatomy, in the University of Pennsylvania—Coroner's Physician of Philadelphia, etc.*

INTRODUCTORY REMARKS—I. Physical properties of Blood. General considerations. Morphology of the blood corpuscles. Oviparous and mammalian blood. Effects of poisons and of disease upon blood ; blood outside of the body ; effects of dessication and moisture ; chemical and spectroscopic tests.—II. Diagnosis between fresh human blood and that of animals. General distinctions. Uniformity of corpuscles in the individual. Measurements ; methods of preparation ; difference between dry and moist preparation regards diameter of corpuscles. Modes of measurements. Eye-piece micrometry ; stage micrometry and photography ; photo-micrographs of corpuscles of various animals. Re-photographing of the same for diagnosis and gross measurements. Table of comparative measurements by the various observers. Gulliver's plate of comparative diagrams of the sizes of corpuscles ; his classification and measurements. Critical review of the various measurements. General conclusions regards diagnosis of fresh blood.—III. Diagnosis of human blood in criminal cases. Liquid blood ; dried blood ; blood-stains ; old and new methods ; liquids employed, methods of preparation ; experiments ; measurements, difference between re-moistened and fresh blood. Conclusions.—IV. Expert testimony upon blood in criminal cases. Abuse of science, perversion of facts. Peculiar cases from personal experience. Suggestions and precautions.—V. Bibliography, "pro" and "contra," classified.

INTRODUCTORY REMARKS.—The present article has been written to record personal observations in the domain of comparative histology of mammalian blood, together with a brief account of what is generally known about the microscopy of blood and blood examinations, in order to elucidate the subject more fully, and to make it intelligible to those not familiar with hæmatology. I made careful inquiry and experiments regarding points disputed in medical legal science, and I present the plain facts and conclusions as obtained from personal studies which extend over a number of years. I submit, however, at the same time the evidence of others upon the same points, such as it is,

*Read before the College of Physicians of Philadelphia.

"pro" and "contra." The literature in this line of observations is not extensive, as far as *original* microscopical studies are concerned.

True, there are numerous works and papers (See "*Index Medicus*") containing quotations from this or that authority on blood, such as the writers—writers often quite distinguished, but who are not microscopists themselves—saw fit to refer to, or had access to.

I sympathize with the author of a text book on Medical Jurisprudence who is not a practical microscopist himself, if he is undecided whose observations on blood he is to accept as correct, and consequently expresses an adverse or guarded opinion; and I fully sympathize with the lawyer or physician who, as a rule, has access only to such books upon this particular subject that have outlived their usefulness, and which only interfere with the cause of science and justice.

The character and nature of expert testimony as regards blood stains in criminal cases will be fully considered and some peculiar incidents from my own practice will be cited.

I do not enter here into the full details of chemical examination of blood, and do not consider at all the spectroscopic analysis, beyond a mere definition of the method, as both are beyond the scope of the present paper. Moreover, chemical and spectroscopic investigations are of subordinate value in the comparative diagnosis of blood; the microscope only can decide the origin and source of any given specimen of blood, while the former can only establish the mere presence of blood.

I append in the bibliography (as complete as was possible) those authors upon blood stains, and upon comparative studies of blood, which might be found useful for references; as will be also the copy of Gulliver's diagram upon the comparative sizes of blood corpuscles which I introduce. (See *Plate IV.*)

The rest of the illustrations in this article are all original, with the exception of one micro-photograph, by Seiler and two by Sternberg. My own photo-micrographs lack in artistic execution. Yet they are quite useful for the study of the comparative sizes of corpuscles, and have been declared satisfactory by expert photographers.

At this place I wish to acknowledge the valuable assistance in measuring daily, for many months, blood corpuscles in several of my legal cases, and making innumerable calculations, to Drs. A. J. Plumer and J. Leffingwell Hatch, and further I wish to thank Drs William Gray, A. J. Plumer, I. W. Blackburn and Mr. French, for assistance in photography and for making drawings. The sixteen micro-photographs were accurately reproduced by the Levytype Autoglyphic Process, through the liberality of the editors of this journal. But particularly do I wish to thank Mr. S. H. Ashbridge, Coroner of Philadelphia, Mr. Thomas J. Powers, his predecessor, as well as the District Attorneys throughout this State, for their kindness in supplying me with much material for study.

I. Physical Properties of Blood.—The blood corpuscles, which form the subject of this paper, constitute, as is well known, the solid portion of the blood, and represent about one-half of the total volume of blood. The blood is an apparently red, viscid liquid of an alkaline reaction, and a specific gravity averaging 1055 in all animals. In its fresh state it has a salty taste, and an odor more or less peculiar to the animal, which is intensified on the addition of sulphuric acid and the application of heat. The blood corpuscles are suspended in a colorless, clear, albuminous liquid, the liquor sanguinis or blood serum, and can be seen only by the aid of the microscope. The specific gravity of the corpuscles alone is said to be 1088, while that of the serum is 1028.

The blood corpuscles, which have been known since Malpighi described them in 1661, are of three kinds: the red and the white corpuscles and the so-called *blood plates*; the latter having quite lately been discovered (in 1878 and 1882) by Hayem, [51], and Bizzozero, [52],* and proven to be the essen-

*In order to avoid frequent foot notes, the reference to authorities will be found in the Bibliography, at the end of this article. The number attached to the names of the authors refers to the number of reference in the Bibliography.

tial element in the clotting of blood, which clotting, under normal conditions, takes place on its immediate removal from the body. For studies upon these blood-plates see Osler, [53], Welch, [54], etc. These blood plates or third blood corpuscles are very numerous (about 1 to every 20 of the blood corpuscles), and are said to differ in quantity and appearance in the various animals, and undoubtedly, on further study, may serve as a diagnostic factor.

The white blood corpuscles exist in a proportion of about 1 to 500 of the red; under normal conditions they have a pale, milky, granular appearance, are spherical in shape when at rest, but are endowed with amœboid motion, by means of which they may assume almost any shape, and are capable of creeping through the tissues into any recess that offers itself. They have nuclei which become more prominent on the addition of acetic acid, or even water. In size the white blood corpuscles have an average diameter of 1–2700 to 1-3000 of an inch, the size being the same in all vertebrates.

White blood corpuscles outside of the blood vessels in masses, are called pus corpuscles. The white blood corpuscles are the progenitors of the red blood corpuscles, and if the transformation to red blood corpuseles is retarded, then there are seen numerous nucleated red blood corpuscles, which are larger than the rest, and are common in certain blood diseases, together with a large increase in the number of white blood corpuscles. In fœtal life these intermediate nucleated red blood corpuscles are quite numerous, paler and more spherical, and may be diagnostic. Genererally, in intra-uterine life the red blood corpuscles are nucleated larger, and show great variation in size. In the normal child or animal, however young, the blood corpuscles are of the same size as in the adult.

Fig. 1. Red and white blood corpuscles.

Normal. Action of Acetic acid.

The red blood corpuscles, which, however, in reality are not red, but yellow (of a lighter or darker hue in the various animals), are in man and mammals circular, biconcave, non-nucleated discs, being thinner at the centre than at the rounded edge. The only exception to this is found in the red blood corpuscles of the llamas and camels, which are oval, but, contrary to oviparous red blood corpuscles, which are oval and nucleated, are without that feature.

As early as 1681 Leewenhoek observed this fact almost simultaneously with his discovery of the compound microscope. The red color of blood is merely an optical delusion, due to refraction of the hæmoglobin of the red blood corpuscles, which, as stated, have really a yellow color, which is readily observed under the microscope.

In size, the mammalian red blood corpuscles vary from 1–2745 of an inch in the elephant to the 1–12325 of an inch in the musk deer (Gulliver), the average diameter of the corpuscles of most animals fluctuating between 1–3000 and 1–5000 of an inch, the thickness of the discs being from $\frac{1}{4}$ to $\frac{1}{3}$ of its diameter. The largest corpuscles are those of the *Amphiuma*, a Louisiana reptile, measuring $\frac{1}{345}$ of an inch in diameter. (Vide details in next section and Plate iv.) The number of red blood corpuscles, according to Malessez, is 4,000,000 to the cubic millimetre in normal human blood. Vierordt and Welcker give 5,000,000 to the c. mm. Some clinicians regard 6,000,000 as the normal number. The goat, which has much smaller corpuscles (half as large as those of man), has 18,000,000 to the c. mm. (Toldt Gewebelehre.) So it appears that the smaller the corpuscles the greater the quantity. The quantity may, however, vary in the blood of different animals as well as in man. In the rabbit (according to Wormley) there are about 3,500,000 per c. mm.

According to Dana, the ratio of the weight of the total bulk of blood to the weight of the body is considered for man to be 1–13, likewise in the dog ; but in the majority of domestic animals it is less ; for instance, the cat, 1–14 ; horse, 1–15 ; rabbit, 1–18 ; guinea pig, 1–19 ; calf, 1–21 ; sheep, 1–24 ; pig, 1–26 ; ox, 1–29.

*Morphology of red blood corpuscles.*—The fact that mammalian blood has no nuclei is no more disputed, the apparent nucleation being due to its biconcavity, on account of which the centre of the corpuscle appears dark in one focus with a light periphery, while in another focus the reverse occurs. Studies of the minute structure of the red blood corpuscles show (Rollet and Kollmann) that they are made up of a protoplasmic stroma which presents itself in the form of a colorless network, the fibres of which are albuminous and easily coagulable ; in the meshes of this network is a fluid, which contains the hæmaglobin. The network of the stroma is attached to an investing membrane or cell wall of the blood corpuscle, if such exist. Although often rejected, the idea of a cell membrane in red blood corpuscles appears from time to time advocated by various observers. If there is a cell wall, it surely must be one possessing great elasticity, as every microscopist knows how soft, slippery and extremely flexible red blood corpuscles are.

I do not think, however, that the red blood corpuscles have a cell wall or membrane, but agree with those who regard it to be only the outer hardened layer of the protoplasm of the corpuscle. In my observations upon the *microscopic* changes produced by the venom of serpents upon blood corpuscles, which I made at the request of Dr. S. Weir Mitchell,* I have often seen, under the microscope, red blood corpuscles fuse into a colloid mass, which can be stretched and drawn out like molasses candy. I described these changes as follows : (See Fig. 2.)

"The blood discs lose their bi-concavity and assume a spherical form, but without parting with their coloring matter. They exhibit also great adhesiveness, arranging themselves into various sized and shaped aggregations. The corpuscles comprising these groups sometimes appear to fuse so that their outlines cannot be determined under the microscope, even by the highest amplification. In addition, the corpuscles seem to soften and acquire a peculiar ductility and capability to be stretched into threads without fracture. By inclining the stage of the microscope, or making gentle pressure upon the cover glass, allowing, thereby, the liquid to flow,

---

* "Researches upon the Venom of Poisonous Serpents," by S. Weir Mitchell and Edward T. Reichert. Published by Smithsonian Institute, Washington, D. C., 1886. Chap. on Pathology, by H. F. Formad, p. 133.

the red blood corpuscles may be seen to elongate themselves into spindle-shaped or even into fine thread-like bodies. (See Fig. 2.) Such masses of corpuscles appear to act like colloid material.

"This remarkable condition I found, in all experiments with venom, to be of only temporary duration. After a short time, which, in about a hundred observations, was found to vary from a few seconds to a quarter of an hour, the apparently homogeneous cell-mass breaks up anew into individual corpuscles of smaller but uniform size, which then continue to be isolated, or in bead-like rows, but remain spheroidal, *i.e.*, do not regain their disc-like, bi-concave shape."

Figure 2. A, normal red blood corpuscles lying flat and in rouleaux. B, the same, having become spherical upon application of crotalus venom, and at C, fused together partly and drawn out into soft string-like bodies ; D, five or more corpuscles fused and stretched out into a thin beaded thread. Magnified 400 diameters.

The above experiments having been made on various animals, prove that the red blood corpuscles have no cell wall, as the venom has no such effect upon cells that are known to have a cell wall.

Under the influence of any liquid reagent of low specific gravity, *e.g.*, water, the flat, bi-concave shape of the corpuscles is lost, from the imbibition of the reagent, and it becomes spherical, and, consequently, reduced in diameter. Even the oval corpuscles of oviparous blood swell up to a spherical form under this treatment, and may be mistaken for mammalian blood. (Vide Gulliver's plate (Plate IV.), 3d figure and xii., 2d figure, and figure 3 in text).

I have, however, never seen any normal red blood corpuscle increased in size by the action of reagents.

Disease is liable to alter the size of corpuscles. The effect of high fever and of exhaustion in diphtheria, tends to diminish the corpuscles, by transforming them into more or less spheroidal bodies with reduction of size.

I had the opportunity to observe this repeatedly with Dr. Horatio C. Wood, in our observations on Diphtheria, for the National Board of Health, during an epidemic of this disease in Michigan and this city.*

The corpuscles fail to form rouleaux in fatal cases of Diphtheria, in consequence of the loss of their bi-concavity. According to Manassein, who made extensive observations at the Military Medical Academy, at St. Petersburg (*Central Blatt fur Med. Wis.*, 1871), the blood corpuscles diminish in size from the effect of high temperature and carbonic acid gas, and he also found that this occurs in Septicæmia.

On the other hand, blood diseases proper are capable of giving rise to an increase in the size of the red blood corpuscles. In pernicious anæmia and chlorosis, this has been observed by Eichhorst. The corpuscles become of uneven size, some are enlarged, while others are diminished below average size. I can corroborate this observation. Further, it is said (Manassein and others), that the red blood corpuscles enlarge from the effects of agents lowering the temperature of the body, such as Alcohol and Quinine. In my own observations in the pathology of Alcoholism, made upon many hundreds of fatal cases, I have found that alcohol has no such effect.

Blood, outside of the body, when *slowly* evaporating or drying, "en masse," shows a diminution in the diameter of the corpuscles ; it never, however, shows an increase, except when a thin layer of fresh blood is dried upon a glass slide, and then this increase is hardly appreciable. I made

*Vide Memoirs on the Nature of Diphtheria. By Drs. H. C. Wood and H. F. Formad. Report of the National Board of Health for 1882.

a series of experiments with blood, regarding the behavior of the corpuscles, as affected by time, under the most varying conditions, to which I will refer at another place. In general, it may be said that rapid desiccation stops all changes in the blood. Blood stains or clots of several years' standing, which had been thoroughly dry from the beginning, showed under proper treatment as perfect corpuscles as dried clots several days old.

Blood which dries slowly does not give good results, and moisture may lead to such degree of decomposition within several days or weeks that no method will enable us to establish the kind of blood; the shape and size of the corpuscles are much altered, and sometimes it is impossible to make out even the outlines of the corpuscles.

Under such circumstances, and when the question relates only to establishing the presence of blood, without regard to its source, the chemical and spectroscopical tests do good service, and can be relied upon.

*Hœmin crystals*, which represent a product of decomposition of the coloring matter of the blood, may be prepared by the addition of glacial acetic acid and sodium chloride to dried blood. A few granules of dried blood are pulverized on a glass slide together with a few granules of salt; having covered it with a glass circle, a drop of the acid is allowed to flow under; the slide is then submitted to heat, when the peculiar crystals appear. (Vide Fig. 3.) The crystals are also known as Teichmann's crystals, after their discoverer, who attributed to them diagnostic properties as regards the blood of different animals. This, however, has not been substantiated by later observations. Hæmin crystals can

Figure 3.

Normal *white* and *red blood corpuscles;* a few are crenated from effect of drying.

*Hæmin crystals* from human blood and a few corpuscles swollen from action of water.

be relied upon as indicating the presence of blood, but cannot be relied upon with certainty as indicating the kind of blood.

*The Guaiacum test* is also an old and a good chemical test. Wormley describes it as follows:

"On treating a solution of the coloring matter of blood with an alcoholic tincture of guaiacum and an ethereal solution of hydrogen peroxide, a deep blue coloration is produced, due to the oxidation of the guaiacum resin. The alcoholic solution should be freshly prepared from inner portions of the resin. The ethereal solution of peroxide of hydrogen, known in the shops as *ozonic ether*, may be prepared by suspending some pure barium dioxide in water, adding an equivalent quantity of dilute sulphuric acid, and extracting the liberated hydrogen peroxide by ether. A portion of the ether extract, if fit for use, will strike a beautiful blue or violet coloration on the addition of a fragment of chromic acid.

"In applying this test, a drop of the blood solution, placed over a white surface or in a porcelain dish, is first treated with a drop of the guaiacum tincture, and then a drop of the ether reagent added, when, even if only a trace of the coloring matter of blood be present, a blue color will immediately or very quickly appear. A drop of a 1–1000th solution of blood will thus immediately yield a decided blue coloration; and a 1–5000th solution a quite distinct reaction.

"The test may be applied directly to the stain, if on a white fabric, by moistening it with a drop of water, and then adding the guaiacum and ethereal solutions. Even the minutest shred of a blood-stained fabric may show this coloration. When the stain is on colored material, it may be as advised by Dr. Taylor, thoroughly soaked with a drop of water, and the liquid absorbed by slips of white bibulous paper; these, while still moist or after they have dried, are submitted to the action of the reagents.

"This test will react even with very old stains, provided they are first well moistened with water; and even when the stains have been washed, evidence of their nature may be obtained."

*The spectroscopic test* for blood consists in passing light through a suspected liquid, and thence through a prism, and if the liquid contains the least trace of blood, certain rays of light will be absorbed, which will cause corresponding dark bands in the spectrum. This is an infallible test for blood, but is also useless when a differential diagnosis as to the kind of blood is to be made, and is superfluous when a single red blood corpuscle can be seen by the microscope ; yet Sorby claims that the delicacy of this test is such that a faint spectrum can be obtained from a single red blood corpuscle. It was introduced by Hoppe-Seyler in 1862. I cannot spare it further attention in this article.

II. Diagnosis Between Fresh Human Blood and that of Animals.—There is nothing by which human blood, "en masse," can be distinguished from that of animals, by the naked eye or by any chemical tests. The differentiation rests entirely upon the microscopical appearance, and chiefly upon the size of the corpuscles. The shape of the corpuscles is diagnostic in distinguishing oviparous blood (non-placental animals) from mammalian (placental animals). The corpuscles of the former have a nucleus, and are invariably oval and more or less convex, whereas the corpuscles of the mammals are devoid of a nucleus and present themselves as round, bi-concave discs ; the only exception from this is the camel species of the ruminantia. (Vide Plate IV., Figs. i, k, l, m, n.)

The red corpuscles of all the mammals (with the exceptions already stated) have absolutely the same appearance morphologically, but differ merely and solely in size.

The distinction, then, whenever it can be made, is only to be established by the measurement of the diameter of the corpuscles.

True, there are certain peculiarities in the hue of the corpuscles. The highest developed animals have corpuscles which are somewhat deeper colored (yellowish red) than those of animals of lower type, and as we descend in the scale of animal life, the corpuscles become of paler yellowish hue, until they are quite colorless as in the amphyoxis. All observers seem to have noticed that the red blood corpuscles of the normal man are, as a rule, the deepest in color, and, as some microscopists express it, "have the stamp of individuality." *

* The corpuscles of the dog appear to have a color next deepest in intensity to man. and it seems that those animals who have corpuscles approaching in diameter those of man, are likewise of a deeper hue ; such is the case in the kangaroo, opossum, and others (Gulliver). The palest in color, among mammals within our reach, are those of the rabbit.

I fully agree with this, after looking nearly every day at numerous specimens of human blood, often side by side with other kinds of blood in the laboratory instruction of my students, for a good many years. Yet, "impressions" do not come into consideration, and should not on the witness stand.

I will return now to the question of distinguishing human blood from that of other animals, by means of micrometry of their corpuscles. I will limit myself exclusively to mammalian blood, as the oval, nucleated blood of the ovipara cannot be confounded with human blood. (See Plate IV.)

*Measurement of Blood Corpuscles.*—Although the average size of corpuscles in the same individual is quite uniform, there are often seen corpuscles which deviate from the average, being either somewhat larger or smaller. The number of such corpuscles is, however, by no means as large as generally held, and their presence mostly due to changes subsequent to the removal of the blood from the body.

Prominent physiologists assert that there are hardly any variations in size among the corpuscles of the same individual while circulating through the living blood vessels. This is in accord with my own observations, and I have found that in microscopic blood preparations, when successful, or at least in one out of ten of them, there will not be more than five to ten corpuscles among one hundred that deviate from the average in any perceptible manner. The distinguished hæmatologist, Hayem, does not admit that there are, in the blood of an individual, more than twelve corpuscles larger, and twelve smaller than the average, among one hundred corpuscles measured. There may be differences in the size of the corpuscles in different individuals of the same species, but these variations are, in my experience, very insignificant, and do not require further consideration unless abnormal conditions come in question. Of some interest in this connection is the paper of Dr. J. G. Richardson,[39], on the identity of the red blood in the different races of mankind.

The average diameter of the human red blood corpuscles is generally given as 1.3250 on an English inch, or 0.0078 mm.

Gulliver,[26], and others, give it as 1–3200, which figures I have adopted as the average, because it better agrees with all the measurements I ever made, or any one of my assistants made.

The variations in the size of individual normal human corpuscles ranges between 1–2900 and 1–3800 of an inch, with but few corpuscles of either extremes, the bulk (90 per cent.) measuring between 1–3100 and 1–3300 in.

The means for establishing the average diameter of the corpuscle in any individual are as follows :

1. By micrometry directly. (See Fig. 4.)

2. By measuring photographic negatives of blood corpuscles mounted upon a stage micrometer.

3. By re-photographing micro-photographs of corpuscles and comparative gross measurements of the amplified photographs. (See Figs. 8, 9, 10, 11 and 12.)

The method generally adopted for preparing blood for micrometric purposes, as well as for photographing, is, to spread it in a thin layer, single layer if possible, upon a glass slide, and dry it rapidly. This is best done by putting a small fresh drawn drop upon a slide and quickly drawing the edge of another slide across the field in such a manner that the corpuscles become evenly distributed ; they may also be spread upon a cover glass. Only those preparations are successful which dry rapidly. Only when immersion lenses are used is it necessary to cover the slide by means of a cover glass. This method has been attributed to Dr. Christopher Johnson, of Baltimore, although it appears that Gulliver and others employed the same method in their earliest observations.

Blood may be examined fresh in its liquid state by putting a minute drop upon a glass slide, covering it with a glass circle, and, in order to prevent evaporation, ringing it with oil or melted paraffine.

From repeated observation I have found that the blood corpuscles prepared by the dry method give diameters slightly larger than those of blood mounted in its liquid state. The reason is that the corpuscles are often more flattened from collapse of the stroma and loss of the bi-

*PLATE I.*

**OX** AND **HUMAN**

FIG. 4. Blood Corpuscles Side by Side, Magnified 500 Diameters. Micrometry Illustrated. Photo-Micrograph by Dr. Seiler.

FIG. 5. FRESH HUMAN BLOOD. Red Blood Corpuscles, Magnified 2250 Diameters. Photo-Micrograph. 1-18 Zeiss Hom Oil Immersion and Projection Eye-Piece.

concave form, which results in a slight increase in their diameter. Yet this increase is scarcely appreciable even by micrometry, though it may account for the variations in the results of measurements. This is also in accord with Gulliver's,[26] * Richardson's,[23], and Masson's,[47] observations. If all the preparations of a series of experiments are treated alike, then the slight spreading of the corpuscles does not affect observation and comparison ; but one should not examine in comparative studies slides made after different modes of preparation.

*Micrometry of Blood.*—The essentials for measuring blood corpuscles consist in a good microscope, provided with a homogeneous immersion lens and two micrometers, one being a stage micrometer, the other one an eye-piece micrometer. The stage micrometer is for establishing the value of the lines on the eye-piece micrometer, and consists of a glass slide ruled to a scale either in mm. or fractions of an inch. The English standard, which I prefer, consists of a series of lines the 1–100 of an inch apart, one of these divisions being further subdivided into thousandths of an inch. In some micrometers smaller divisions have been resorted to.

There are occasionally great errors in the ruling of these micrometers, so that they should be tested before using. This may be done under a high power, comparing one division with another, and noting any discrepancy which may occur. If there should be an error in the size of some of the divisions, then it must be determined which are exact, and only such used for a standard. Discrepancies as much as the 1–40,000 of an inch have been known to occur.

The eye-piece micrometers are of various kinds, but all are on the same plan, which is a slip of glass, with fine lines ruled to a uniform scale, and which fits in the eye-piece of the microscope. When placed in position its value is determined by the aid of the

* "The corpuscles of man and other mammals, when dried on glass, however quickly, they were usually just appreciably larger than in the liquor sanguinis, as if they were slightly spread out and prevented from as slight a contraction in sticking to the object plate."

stage micrometer, noting how many of the divisions on the eye-piece micrometer are required to fill one of the divisions on the stage micrometer. Suppose that we employ a 1 12 Zeiss hom. mer. lens, and that under this amplification the 1–1000th of an inch division of the stage scale covers exactly twenty places in the eye-piece scale, then each division of the eye-piece micrometer will be equal to the 1–20,000th of an inch. Higher objectives will increase the value of the divisions, lower ones will decrease them, but under all circumstances the same conditions must be preserved, which are well known to those familiar with the technique of microscopy, which is beyond the scope of this paper. Care must be taken that the draw tube of the microscope be always in the same position, and frequent control with stage micrometer must be exercised. The glass slides used must always be of the same thickness and the glass covers of the thinnest sort.

To apply this method in practice simply requires to bring a field of blood into focus under the eye-piece micrometer, previously adjusted, and observe the number of divisions or fraction of a division of the eye-piece micrometer that a corpuscle may occupy. See Fig. 4, Plate I.) For instance, if a corpuscle should exactly fill four spaces, then its value under a 1–20,000th of an inch, standard, would be $\frac{4}{20,000}$ or $\frac{1}{5,000}$ of an inch. In this manner a number—not less than a hundred—of measurements should be made, and from different slides, and the average taken as the result.

All measurements should be made of perfect round biconcave corpuscles only, and should be carefully recorded. Abnormally small and crenated (shrivelled) blood corpuscles should be avoided.

*The micrometry of blood corpuscles from photographic negatives* has been introduced by Dr. Carl Seiler,[31] of Philadelphia, and soon after followed by Dr. Woodward,[35] of U. S. A. The plan is simply to mount the blood directly upon a glass stage micrometer and to photograph then with any desired amplification, both blood and micrometer appearing sharply defined on the picture. The measurements are then made directly on the negative. Dr. Woodward says that he employed a professional photographer to execute the technique of photography.

It is necessary to understand that the micrometry of blood corpuscles is expressed by various observers in various scales. On the continent of Europe the metric system is exclusively employed, whereas the English and American hæmatologists are in the habit of expressing the measurements in fractions of an inch, *e.g.*, in the French system the diameter of the human corpuscle is 0.0079 mm., which corresponds in the English system to 1–3200 in.

Most authorities expressing themselves in mm. carry the fractions out only four places; in fact, this is the uniform custom, but is not nearly so accurate as the fraction of an inch with four figures in the denominator.

I consequently prefer the English system, and only introduce the French system for the convenience of those who are more accustomed to it. In the table of measurements I have placed the inches side by side with the millimetres. Frequently, however, the French employ a vulgar fraction of a mm., and on the other hand, the English a decimal of an inch; so that the average diameter of human blood corpuscles is expressed as $\frac{1}{126}$ of a mm. by the former and .000313 of an inch by the latter, which is equal to 0.0079 mm., and to 1–3200 of an inch.

Although this variety of expressing differently one and the same thing can be easily reconciled by means of a little arithmetic, it has, in some instances, led to errors and misunderstandings.

Errors have also occurred from the divergency of the results caused by the different modes of micrometry.

A striking example is this: Measuring blood corpuscles upon photographic negatives, taken by the method described above, has led Dr. Woodward[35] into the error of giving the micrometry of the Guinea pig's blood as 1–3213 in., and this has induced other (later) observers to give a similar figure, or figures near this. If his figure of the Guinea pig's blood corpuscles, 1–3213 in., be correct, then his figures for the human and dog's corpuscles respectively, made upon the same scale, should be correct ; but we find them to be far from the accepted average figures ; Woodward's figures being for the corpuscles of man 1–3092 of an in., and for those of the dog 1–3246 of an in. Here is evidently an error, because, if Woodward's figure given for man is wrong, then his figures for the Guinea pig and the dog are wrong. If, however, Woodward's figures for the human corpuscle be lowered to the correct figure of 1–3200, then the Guinea pig and the dog, correspondingly lowered, will prove to have their rightfully sized corpuscles, viz.: 1–3300 to 1–3500 of an inch, all three figures being made upon the same scale. Furthermore, Dr. Woodward's micrometry of the Guinea pig's blood was very insufficient, he having examined only 401 corpuscles, all from one drop of blood, and a single individual. The species of Guinea pig examined by Woodward was the ordinary (*cavia cobaya*). Other animals he did not examine.

To substantiate my objections, and in order to show how the mistake arose, let us analyze Dr Woodward's[34] results: His measurements were expressed in millionths of an inch, and millionths of a millimetre, and in the case of the human blood, were made from 22 negatives, taken from 9 drops of blood, obtained from 8 individuals, the whole number of corpuscles measured being 1766. The average result of all these measurements was .000323 of an in., or .008214 mm., when transferred to the usual form, give the impossible figures 1–3092 in., or 0.0082 mm. for the average diameter of the human red blood-corpuscles.

His measurements of the corpuscles of the dog were calculated from 13 negatives, prepared from 5 drops of blood, each taken from a single individual, the whole number of corpuscles measured being 1,571, and yielded an average of .000308 of an in., or 0.007823 mm. These, if transferred like above to the usual figures, give us 1–3246 of an in., or 0.0078 mm., which erroneously brings it to the average of the human corpuscle. He measured 401 corpuscles of the Guinea pig from 4 negatives, prepared as stated from one drop taken from a single individual, the average obtained being .000311 of an in., or 0,007905 mm., which, transferred to the usual

PLATE II.

FIG. 6. BLOOD CORPUSCLES OF MAN, Magnified 1450 Diameters.

FIG. 7. BLOOD CORPUSCLES OF GUINEA PIG, Magnified 1450 Diameters.

Micro-Photographs by Dr. Sternberg. 1-18 Zeiss Hom Immersion.

Both Photo-Engravings are slightly but uniformly enlarged.

expression, gives rise to the misleading figures, 1–3213 of an in., or 0.0079 mm.

The results of the micrometry of Professors Wormley[46] and of Masson[47] closely approach the figures of Woodward[35] for the Guinea pig. Wormley, who examined 300 corpuscles of one wild Guinea pig (*cavia aperea*), gives an average diameter of 1–3223 in., while Masson's measurements, limited also to one individual (using, however, the *cavia cobaya*, the ordinary Guinea pig), was 1–3300 in., or 0.0077 mm. These results of micrometry find also a telling support in the excellent Photo-micrographs of Dr. Sternberg,[55] which I reproduce on Plate II. It does thus appear that the corpuscles of the Guinea pig are brought in a range with those of man, so close that it would seem hopeless to try to tell these two kinds of blood apart.

My own measurements of the Guinea pig's red blood corpuscles gave an average diameter of $\frac{1}{3400}$ in. I examined the *cavia cobaya*, the only accessible Guinea pig (the other, *cavia aperea*, being only a rare menagerie animal).

I examined at different times ten Guinea pigs, making from each ten preparations, and measuring 100 corpuscles from each animal.

The mean of every 1,000 corpuscles was 1–3400 of an inch.

Outside of the systematic observations I have often examined smaller series of blood preparations from this animal with nearly uniform and similar results. The same micrometric results of the Guinea pig's blood (1–3400 in.) were also obtained independently by Drs. J. L. Hatch, A. J. Plumer and Henry Wile, in my laboratory, and they are also in accord with Dr. Richardson's measurements, made in my presence.

Gulliver's figures for the average diameter of the corpuscles of the Guinea pig (*cavia cobaya*), obtained from innumerable measurements, is 1–3538 in. (See table page 275.)

I make systematic measurements of the red blood corpuscles of men frequently; thousands of these measurements I have occasion to do while instructing students and physicians who come to my laboratory for special study.

My measurements were made with an eye-piece micrometer, standardized by a stage micrometer, which had also been compared with a micrometer in the Army Medical Museum, through the kindness of Dr. William Gray. The objective I used was a Zeiss $\frac{1}{12}$ hom. oil immersion, the instrument itself being a Zentmyer's standard. The magnifying power employed is always so fixed that each division of the eye-piece micrometer was equal to the 1–20000 part of an inch. A micrometer of 1–15000 part of an inch I found also quite satisfactory for comparative studies.

Regarding blood corpuscles of creatures below man, my measurements were limited to the domestic animals and the wolf and Guinea pig, *i.e.*, the blood of such animals as on different occasions, in my personal experience with criminal cases, had been (except the Guinea pig) claimed as the source of blood stains upon various articles of apparel, etc. I propose to publish at another occasion some tables and figures of the individual series of measurements, which occupy 82 pages, and are not devoid of interest, but too bulky for this article. The figures I give below represent the average of all the averages of measurements made at different times upon different individuals.

I have adopted as the average for human red blood corpuscles, Gulliver's figure, 1–3200 of an inch. This average figure is nearly uniformly obtained if all accidentally disfigured and unusually (abnormally) small corpusles are excluded from the measurements, as they should be ; moreover, they, as well as "large" corpuscles, are few in number in successful preparations. We have seen that red blood corpuscles often really or apparently diminish in size, but they never (in normal blood) expand above normal size.

This point is very important in the consideration of criminal cases. Possible mistakes in the diagnosis of the origin of blood "might contribute to a criminal's escape, but *never* to the punishment of the innocent party," as we will see later.

The following is the mean of my own measurements of the red blood corpuscles of the several animals measured in the manner described above :

| | | |
|---|---|---|
| Man | 1-3200 | of an inch |
| Guinea pig | 1-3400 | " |
| Wolf | 1-3450 | " |
| Dog | 1-3580 | " |
| Rabbit | 1-3662 | " |
| Ox | 1-4200 | " |
| Pig | 1-4250 | " |
| Horse | 1-4310 | " |
| Sheep | 1-5000 | " |
| Goat | 1-6100 | " |

*Comparative Study and Measurements of Red Blood Corpuscles, by Means of Photography.*—A very convenient method of plainly demonstrating the difference in the sizes of the blood corpuscles I found to be the gross measurements of photographs of corpuscles, enlarged to the size of 10,000 diameters. Under such amplification the absolute difference in the diameter of corpuscles becomes quite perceptible to the naked eye

Such amplification of blood corpuscles can, of course, only be obtained by re-photographing single corpuscles from photo-micrographs, selecting such that are of average size. The method I adopted is as follows: I first take photo-micrographs of fresh blood (prepared in the usual manner) of man, dog, Guinea pig, ox, sheep and goat, all executed under the same amplification and projection, and under absolutely similar conditions. Then I have positives prepared from each, and enlarging now single corpuscles of average size, selected from each positive, by re-photographing, I obtained admirable results. In order to get at the comparative size of corpuscles, I selected one human blood corpuscle of 1-3200 inch, and enlarged it by photography to the size of 3⅛ inches, which represents the desired amplification of 10,000 diameters. (See Fig. 8, Plate III.) Any other corpuscle of 1-3200 in. will of course be 3⅛ in. under the same projection; but substituting now, for that of man, the positives of corpuscles of the dog, Guinea pig, ox, sheep and goat seriatim, and of known micrometry, re-photographing each separately, but all under absolutely the same projection, distance and focus, the striking difference in the size of the corpuscles is quite apparent.

Such corpuscles of each animal which represent the average size having been selected, the photographic amplification gives such results as are seen in Figs. 8, 9, 10, 11, upon Plate III. (page 273). Whereas, the difference in the sizes of blood corpuscles is often not quite obvious under low amplifications, it becomes very apparent and evident when enlarged to such magnitude by means of simple photography. The difference between 1-3.00 in. (man) and 1-3400 (guinea-pig) is only 1-54,400 inch. When magnified 10,000 diameter, this difference is equal to nearly 1-6 of an inch. It is out of place to speak here on the technique of photography. I always prefer to have the assistance of an expert photographer ; this leads to good results and saves much time.

When thus magnified to 10,000 diameters, we find that the corpuscles measure as follows :

| | | | |
|---|---|---|---|
| Human 1.3200 of an inch actual micrometry) | | $=3\frac{1}{8}$ | inches. |
| Guinea pig | (1–3400) | $=3$ | " |
| Dog | (1–3500) | $=2\frac{4}{5}$ | " |
| Ox | (1–4200) | $=2\frac{1}{3}$ | " |
| Sheep | (1–5000) | $=2$ | " |
| Goat | (1–6100) | $=1\frac{3}{5}$ | " |

The first column of figures indicates the actual size of the selected corpuscles ; the second gives the gross measurement of the same corpuscles when amplified 10,000 diameters and the photographic image measured (upon the negative) with an ordinary gross carpenter's tape-measure.*

Such illustrations of the comparative sizes of the red blood corpuscles I found to be very convenient for use in lectures and for general demonstration of the subject on the witness stand. I do not entirely rely upon photo-micrography for the direct and absolute measurements of corpuscles, but I do consider photography applied by my own method as the best and surest means for establishing the relative or comparative diameter of corpuscles when the question arises to decide between two or more kinds of blood.

---

* Measurements are to be made upon the negatives. Printed photographs are not reliable for measurements on account of the stretching of the paper.

### COMPARATIVE TABLE OF THE AVERAGE RESULTS OF MEASUREMENTS OF RED BLOOD CORPUSCLES OF MAMMALS.

Each column giving the average size (diameter) of the Corpuscles as obtained by various observers, expressed in fractions of the English inch, side by side with the common expression (roughly) in millimeters.

| | Gulliver, 1845 and 1875. | | Wormley, 1885. | | C. Schmidt, 1848. Mallinin, 1875. | | French Medico Legal Society, 1873, and Welker. | | Masson, 1885. | | Hans Schmid, 1878. | | Woodward, 1875. | | Personal Observations. | |
|---|---|---|---|---|---|---|---|---|---|---|---|---|---|---|---|---|
| | In. | M. M. | In. | M. M. | In. | M. M. | In. | M. M. | In. | M. M. | In. | M. M. | In. | M. M. | In. | M. M. |
| *Elephant* | 1.2745 | 0.0092 | 1.2738 | 0.0093 | ... | ... | ... | ... | ... | ... | ... | ... | ... | ... | ... | ... |
| *Great Anteater* | 1.2769 | 0.0088 | ... | ... | ... | ... | ... | ... | ... | ... | ... | ... | ... | ... | ... | ... |
| *Walrus* | 1.2769 | 0.0088 | ... | ... | ... | ... | ... | ... | ... | ... | ... | ... | ... | ... | ... | ... |
| *Sloth* | 1.2865 | 0.0086 | ... | ... | ... | ... | ... | ... | ... | ... | ... | ... | ... | ... | ... | ... |
| *Ornithorynchus* | 1.3000 | 0.0081 | ... | ... | ... | ... | ... | ... | ... | ... | ... | ... | ... | ... | ... | ... |
| *Whale* | 1.3099 | 0.0080 | ... | ... | ... | ... | ... | ... | ... | ... | ... | ... | ... | ... | ... | ... |
| *Opossum* | 1.3557 | 0.0071 | 1.3145 | 0.0080 | ... | ... | ... | ... | ... | ... | ... | ... | ... | ... | ... | ... |
| *Capybara* | 1.3190 | 0.0080 | 1.3164 | 0.0080 | ... | ... | ... | ... | ... | ... | ... | ... | ... | ... | ... | ... |
| *Man* | 1.3200 | 0.0079 | 1.3250 | 0.0078 | 1.3300 | 0.0077 | 1.3257 | 0 0078 | 1.3257 | 0.0078 | 1.3412 | 0.0074 | 1.3092 | 0.0082 | 1.3200 | 0.0079 |
| *Seal* | 1.3281 | 0.0078 | ... | ... | ... | ... | ... | ... | ... | ... | ... | ... | ... | ... | ... | ... |
| *Beaver* | 1.3325 | 0.0076 | ... | ... | ... | ... | ... | ... | ... | ... | ... | ... | ... | ... | ... | ... |
| *Muskrat* | 1.3550 | 0.0072 | ... | ... | ... | ... | ... | ... | ... | ... | ... | ... | ... | ... | ... | ... |
| *Porcupine* | 1.3369 | 0.0075 | ... | ... | ... | ... | ... | ... | ... | ... | ... | ... | ... | ... | ... | ... |
| *Monkey* | 1.3412 | 0.0074 | 1.3382 | 0.0075 | ... | ... | ... | ... | ... | ... | ... | ... | ... | ... | ... | ... |
| *Kangaroo* | 1.3440 | 0.0074 | 1.3410 | 0.0074 | ... | ... | ... | ... | ... | ... | ... | ... | ... | ... | ... | ... |
| *Guinea Pig* | 1.3538 | 0.0071 | 1.3223 | 0.0079 | ... | ... | ... | ... | 1.3300 | 0.0077 | ... | ... | 1.3213 | 0.0079 | 1.3400 | 0.0075 |
| *Wolf* | 1.3600 | 0.0070 | 1.3422 | 0.0074 | ... | ... | ... | ... | ... | ... | ... | ... | ... | ... | 1.3450 | 0.0074 |
| Dog | 1.3532 | 0.0071 | 1.3561 | 0.0071 | 1.3636 | 0.0070 | 1.3485 | 0.0073 | 1.3577 | 0.0071 | 1.3846 | 0.0066 | 1.3246 | 0.0078 | 1.3580 | 0.0071 |
| Rabbit- | 1.3607 | 0.0070 | 1.3653 | 0.0070 | 1.3968 | 0.0064 | 1.3653 | 0.0069 | 1.3636 | 0.0070 | ... | ... | ... | ... | 1.3662 | 0.0069 |
| Ass | 1.4000 | 0.0063 | 1.3620 | 0.0070 | ... | ... | ... | ... | ... | ... | ... | ... | ... | ... | ... | ... |
| Rat | 1.3754 | 0.0068 | 1.3652 | 0.0070 | 1.3968 | 0.0064 | ... | ... | ... | ... | 1.5000 | 0.0051 | ... | ... | ... | ... |
| Bear | 1.3693 | 0.0070 | 1.3656 | 0.0069 | ... | ... | ... | ... | ... | ... | ... | ... | ... | ... | ... | ... |
| Mouse | 1.3814 | 0.0067 | 1.3743 | 0.0067 | 1.5000 | 0 0051 | ... | ... | ... | ... | ... | ... | ... | ... | ... | ... |
| Mule | ... | ... | 1.3760 | 0.0067 | ... | ... | ... | ... | ... | ... | ... | ... | ... | ... | ... | ... |
| Squirrel | 1.4000 | 0.0064 | 1.4140 | 9.0061 | ... | ... | ... | ... | ... | ... | ... | ... | ... | ... | ... | ... |
| Ox | 1.4267 | 0.0060 | 1.4219 | 0.0060 | 1.4354 | 0.0058 | 1.4545 | 0.0056 | 1.4237 | 0.0060 | 1.4695 | 0.0054 | ... | ... | 1.4200 | 0.0060 |
| Pig | 1.4230 | 0.0060 | 1.4268 | 0.0059 | 1.4098 | 0.0062 | 1.4098 | 0.0062 | 1.4098 | 0.0062 | 1.4098 | 0.0062 | ... | ... | 1.4250 | 0.0060 |
| Horse | 1.4600 | 0.0055 | 1.4243 | 0.0059 | 1.4464 | 0.0057 | 1.4545 | 0.0056 | ... | ... | ... | ... | ... | ... | 1.4310 | 0.0059 |
| Cat | 1.4404 | 0.0058 | 1.4372 | 0.0058 | 1.4545 | 0.0056 | 1.3922 | 0.0065 | 1.4440 | 0.0057 | ... | ... | ... | ... | ... | ... |
| Sheep | 1.5300 | 0.9048 | 1.4912 | 0.0951 | 1.5649 | 0.0045 | 1.5076 | 0.0050 | 1.4464 | 0.0057 | 1.6060 | 0.0042 | ... | ... | 1.5000 | 0.0051 |
| Goat | 1.6366 | 0.0040 | 1.6189 | 0.0041 | 1.6369 | 0.0040 | 1.5525 | 0.0046 | ... | ... | ... | ... | ... | ... | 1.6100 | 0.0042 |

This table is an accurate compilation of the average measurements of the red blood corpuscles of mammals, as given by the various original observers, each being represented by a separate column. All other tables of measurements, as given in books, are according to some one of the authorities quoted in this table, some of whom are not accessible to the casual reader.

For convenience, I have put the English fraction of an inch and the French millimetre, side by side; the former being transformed into the latter, or the latter into the former, as the case required, by Drs. J. L. Hatch and A. J. Plumer, and my brother, Dr. R. Formad, Jr., Veterinarian.

A study of this table will show its usefulness; it illustrates and elucidates many interesting points. It shows that the results obtained by the various observers, as regards the micrometry of the blood corpuscles of the majority of animals, is remarkably uniform, and that some of the measurements made by Gulliver,[26] with imperfect instruments, nearly fifty years ago, are in accord with those made with the more perfect instruments of the present day. (In fact, Gulliver states that Jurin, 150 years ago, estimated the human blood corpuscles as 1-3240 of an inch in diameter.) On the other hand, the table shows there is quite a discrepancy as regards the diameters of the corpuscles of some animals, so that it entirely depends upon whose figures we accept, whether we can or cannot discriminate between the human blood and the blood of certain animals.

The most extensive measurements are those of Gulliver. I have given in this table only a small part of his work, viz.: that relating to measurements of the corpuscles of those animals which have a more or less peculiar interest. The total number of his measurements embraces nearly 800 animals, and extended over thirty-five years. He made them solely from a biological standpoint, claiming that the blood corpuscles are one of the prominent means of the classification of animals into species.

Gulliver is the pioneer in hæmatology, and it may be interesting to note his own opinion regarding micrometry. He says: "My tables cannot pretend to absolute exact-

*PLATE IV.*

**Gulliver's micrometry of red blood corpuscles, all to a uniform scale.**

G. Gulliver ad. nat. del.

ness, and are only offered for what they may be worth; and in the estimation of their value, allowance should be made for errors, whether instrumental or personal, more or less inevitable, notwithstanding the greatest care, in observations so extensive." "Nevertheless," he adds, "the relative value of the measurements, though probably not unexceptionable, may be entitled to more confidence as fair approximation to the truth."

No doubt, the comparative relations of the sizes of the corpuscles are given correctly by Gulliver; accurately enough for scientific as well as all practical purposes. They are nearly uniformly in accord with all later measurements. Gulliver made his measurements both from fresh blood and from blood thinly smeared and dried upon a glass slide, precisely by the same method as is generally used at present.

A most convenient and beautiful illustration of the differ ence in the sizes of red blood corpuscles of the various animals, is Professor Gulliver's plate, which I here reproduce. The diagrams upon this (Gulliver's) plate give a most excellent idea of the comparative diameters of blood corpuscles in *fresh* blood.

I reproduce also Gulliver's own explanation of the plate and the classification of the animals, which is quite interesting from a biological standpoint, and it explains itself. It gives the measurements of the diameters of corpus cles of a number of animals that are not incorporated in my table of comparative observations. Both plate and explanation of plate (page 278) are accurate copies from Gulliver's famous article; I only added the English names to the Latin denominations. (Some oversights of the engraver as regards omission of figures on the plate are explained in the text.)

*Explanation of the figures upon Gulliver's plate.*—All the objects are red blood corpuscles done to one and the same scale, which is at the foot of the drawing. The whole length of the scale represents 1–1000 of an English inch, and each one of the ten divisions 1–10,000 of an inch. Only corpuscles of the average sizes and quite regular shapes are given; and they are all magnified to the same, to wit, about 800 diameters. For details see description below.*

*It seems to be 900.—H. F. F.

A.—VERTEBRATA APYRENÆMATA. (See Plate IV.)

I. Homo (Man) .......... 1–3200

*1. Corpuscles lying flat.

2. The same on edge.

3. Membraneous base of same after removal by water of coloring matter; it shows diminution in diameter on account of acquired spherical shape.

II. Quadrumana (Monkeys.)

4. Simia troglodytes (Chimpanzee) .......... 1–3412

5. Ateles ater. (Black-faced spider monkey) .......... 1–3602

6. Lemur anguanensis .......... 1–4003

III. Cheiropetera (Bats.)

7. Cynonycteris collaris (fruit bat) .......... 1–3880

8. Vespertilio noctula (large bat) .......... 1–4404

9. Vespertilio pipistrellus (common bat) .......... 1–4324

IV. Feræ (beasts of prey.)

(p) 10. Sorex tetragonurus (shrew) .......... 1–4571

(q) 11. Ursus labiatus (lipped bear) .......... 1–3728

(r) 12. Bassaris astuta civet cat) .......... 1–4033

(s) 13. Cercoleptes caudivolvulus (kinkajou) .......... 1–4573

(t) 14. Trichechus rosmarus (walrus) .......... 1–2769

(u) 15. Canis dingo (dog, Australian) .......... 1–3395

(w) 16. Mustella zorilla (weasel) .......... 1–4270

(a) 16. Felis leo (lion) .......... 1–4322

(b) 16. Felis leopardus (leopard) .......... 1–4319

(x) 17. Felis tigus (tiger) .......... 1–4206

(y) 18. Paradoxurus pallasii (Pallas paradoxure) .......... 1–5485

(z) 19. Paradoxurus bondar (Bondar Paradoxure) .......... 1–5693

(a) 19. Hyena striata (striped hyena) .......... 1–3735

V. Cetacea. (Whales.)

20. Balæna (boops) (whale) .......... 1–3099

21. Delphinus globiceps (caing—whale) .......... 1–3200

22. Delphinus phocæna (porpoise) .......... 1–3829

VI. Pachydermata.

23. Elephas indicus (elephant) .......... 1–2745

24. Rhinoceros indicus (rhinoceros) .......... 1–3765

25. Tapirus indicus (tapir) .......... 1–4000

26. Equus caballus (horse) .......... 1–4600

27. Dicotyles torquatus (peccary) .......... 1–4490

28. Hyrax capensis (Cape hyrax) .......... 1–3308

VII. Ruminantia (Ruminants.)

(a) 29. Tragulus javanicus, (Javan chevrotain, musk deer) .......... 1–12325

*Through an oversight, some of the figures are not marked upon the plate.

| | | |
|---|---|---|
| (b) 30. Tragulus meminna (Indian chevrotain) | | 1–12325 |
| (c) 31. Tragulus Stanleyanus (Stanleyan chevrotain) | | 1–10825 |
| (d) 32. Cervus nemorivagus (deer) | | 1–7060 |
| (e) 33. Capra Caucasica (Caucasian ibex) | | 1–7045 |
| (f) 34. Capra hircus (domestic goat) | | 1–6366 |
| (g) 35. Bos urus (represented by Chillingham cattle) | | 1–4267 |
| (h) 36. Camelopardalis giraffa (giraffe) | | 1–4571 |
| (i) 37. Auchenia vicugna (vicuna) | L. D. | 1–3555 |
| | Sh. D. | 1–6587 |
| (k) 38. Auchenia paca (alpaca) | L. D. | 1–3361 |
| | Sh. D. | 1–6229 |
| (l) 39. Auchenia glama (llama) | L. D. | 1–3361 |
| | Sh. D. | 1–6229 |
| (m) 40. Camelus dromedarius (single hump camel | L. D. | 1–3254 |
| | Sh. D. | 1–6931 |
| (n) 41. Camelus bactrianus (double hump camel) | L. D. | 1–3123 |
| | Sh. D. | 1–5876 |
| (o) 42. Cervus Mexicanus* (deer—Mexican) | | 1–5175 |

VIII. Rodentia (Rodents).

| | |
|---|---|
| 43. Hydrochœrus capybara (capybara) | 1–3190 |
| 44. Castor fiber (beaver) | 1–3325 |
| 45. Sciurus cinereus (squirrel) | 1–4000 |
| 46. Mus messorius (harvest mouse) | 1–4268 |

IX. Edentata.

| | |
|---|---|
| 47. Myrmecophaba jubata (ant eater) | 1–2769 |
| 48. Bradypus didactylus (sloth) | 1–2865 |
| 49. Dasypus villa (armadillo) | 1–3315 |

X. Marsupialia.

| | |
|---|---|
| 50. Phascolomys (wombat) | 1–3456 |
| 51. Hypsiprymnus setosus (kangaroo rat) | 1–4000 |

XI. Monotremata.

| | |
|---|---|
| 52. Echidna histrix (echidna) | 1–3840 |

B.—VERTEBRATA PYRENÆMATA.

| XII. Aves (Birds) | L. D. | Sh. D. |
|---|---|---|
| 1. Struthio camelus (ostrich) | 1–1649 | —1–3000 |
| 2. The same made round and deprived of color by water. | | |
| 3. Vanga destructor (East India shrike) | 1–2019 | —1–3892 |
| 4. Lanius excubitor (great grey shrike) | 1–1989 | —1–5325 |
| 5. Bubo virginianus (horned owl) | 1–1837 | —1–4000 |
| 6. Syrnea nyctea (snowy owl) | 1–1555 | —1–4042 |
| 7. Columba rufina (rufous pigeon) | 1–2314 | —1–3329 |
| 8. Columba migratoria (wild pigeon) | 1–1909 | —1–4626 |
| 9. Dolichonyx oryzivorus (rice bird) | 1–2400 | —1–4167 |

* The only animal in which the red blood corpuscles present a variety of shapes in the same individual.—Gulliver.

| | | |
|---|---|---|
| 10. | Buceros rhinoceros (rhinoceros hornbill)... | 1-1690—1-3230 |
| 11. | Psittacus augustus (August amazon)..... | 1-2085—1-3606 |
| 12. | Phasianus superbus (barrel-tailed pheasant) | 1-2128—1-3587 |
| 13. | Pelecanus onocrotalus (white pelican)..... | 1-1777—1-3369 |
| 14. | Trochilus sp. (humming bird)............. | 1-2560—1-4000 |

Figures XII., XIV., XVI., XVII. and XVIII. represent red blood corpuscles of Reptilia and Bactrachia; while under figure XIX. those of the fishes are given. In all these figures the names of the animals are inserted upon the plate, and they do not require any comment at this place. It is evident that the blood corpuscles of the Amphiuma are so large that they can be perceived by the naked eye.

Carl Schmidt,[7] the Russian pioneer hæmatologist (Dorpat, 1848), although making his measurements of blood corpuscles with an amplification of only 500 diameters and drawing his averages from only forty measurements of the corpuscles of each individual, furnished accurate micrometric figures of diameters of blood corpuscles. In fact, they are, with but few exceptions, in accord with Gulliver's (1842) and with measurements made of late years. Carl Schmidt is the father of micrometry as applied to blood stains in criminal cases (1840). It is peculiar that he made most of his measurements of corpuscles from dried blood; cutting thin sections, by means of a razor, from blood clots and examining them in oil. But he, as well as Gulliver, also used our "modern" method of spreading single layers of blood corpuscles on glass slides and drying them rapidly.

From measurements of his own he showed that there was a reliable mean average of the diameter of the blood corpuscles of the different mammals, which could be used for diagnosis in criminal cases. He showed also, that if there was a shrinkage in the size of the corpuscles from drying, this should not debar us from an accurate diagnosis, because he established that the shrinkage was proportionately uniform in the blood corpuscles of all animals. His figures, quoted in my table, show that the latter proposition is correct, since all his measurements are somewhat less than those of other observers, but in a constant, uniform ratio, making them correspond proportionately with the others.

Wormley,[46] Professor of Chemistry in the University of

Pennsylvania, has furnished a good article on the examination of blood, in the appendix of his work on the Micro-Chemistry of Poisons. I have quoted Prof. Wormley's figures of average measurements, which extended over forty-six different animals (thirty-eight mammals, four birds and four reptiles), in my table of comparative measurements. See page 275. On the whole, they are practically in accord with Gulliver's and Carl Schmidt's measurements, and absolutely correct as far as they went. Wormley disagrees essentially with Gulliver only in regard to the measurements of the opossum and guinea pig (see table); but, as he explains, the species of the animals examined in the latter case was not the same.

The late J. G. RICHARDSON,[19 23] whose researches on blood, first published in 1869, have been followed with interest by medical jurists and biologists the world over, was unquestionably the most reliable and most prominent American hæmatologist. He was the first to employ and to advocate such high microscopic objections as 1-25 and 1-50 in the diagnosis of blood corpuscles. Under an amplification of 3,700 diameters obtained with a 1-50 immersion objective, Dr. Richardson found an average-sized human corpuscle to measure $\frac{9}{8}$ of an inch in diameter, while that of a sheep was only $\frac{5}{8}$ of an inch across; an ox blood corpuscle measured $\frac{7}{8}$ of an inch. Thus magnified, the comparative difference was quite apparent. I have often assisted Dr. Richardson in measuring blood corpuscles in his medico-legal cases, and profited much by learning his methods of measuring with high power. He was one of the most prominent advocates of a positive diagnosis of human blood from that of *all* domestic animals by means of micrometry under high amplification. His observations in this field of study are extensive and well known. They are in accord with nearly all later observations, and will be referred to later on.

Masson[47] is the most recent of observers on the measurement of blood corpuscles. He thinks it difficult if not impossible to distinguish the blood corpuscles of man from those of the guinea-pig. From the results of his measurements he found the average diameter of the corpuscles of the guinea-pig to be 1-3300 inches (.0077 mm.). He measured also the blood corpuscles of the rabbit, dog, ox, pig and sheep, with results quoted in my general table. With regards to all these animals, however, he expresses the positive opinion that the blood of neither of those animals can be mistaken for that of man when careful measurements of the corpuscles are made. He says : "One can distinguish with certainty the blood of man and guinea-pig from that of the dog and rabbit, and the blood of the last two named animals from that of the pig, ox and cat."

Lacour[48] published his observations last year ; his results were identical with those of Masson. He asserts that if the average diameter of the blood corpuscles exceeds 1-127 of a mm. (1-3225 of an inch), then the blood is either human or that of the guinea-pig ; but if the diameter is less than 1-127 of a mm., then the blood may be that of the dog or rabbit. If less than 1-135 of a mm., then the blood is not human, nor that of the guinea-pig, rabbit or dog, and if less than 1-400 mm. it may pertain to the ox, pig, sheep, etc.

*General Résumé.*—From all the studies referred to so far, it can be regarded as established, that the microscopist has ample and sure means to diagnose fresh or well-preserved human blood from that of certain animals, provided he has the proper experience and employs rightful and honest means. Surely, human blood can be told from that of all the ordinary domestic animals, not counting the guinea-pig as a domestic animal. It depends, however, whose figures are accepted for the mean diameter of this animal's corpuscles, whether guinea-pig's blood may be mistaken for human. All the animals, whose blood corpuscles closely approach in diameter those of man, are wild, or menagerie animals, and the micrometry of their blood corpuscles has no other but a purely biological interest, unless when improper use is made of it in the defense of criminals.

Strictly speaking, only the following animals have corpuscles larger than man, *i.e.*, larger than 1-3200 of a inch: Elephant, great ant-eater, walrus, sloth, platypus, whale, capybara, and (according to Wormley) opossum. Animals, the corpuscles of which are slightly below man in size, *i.e.*, having corpuscles from 1-3500 to 1-3200 of an inch average diameter, are the seal, beaver, musk-rat, porcupine, monkey, kangaroo, wolf and guinea-pig. (See table page 275.) None of these are domestic animals. All other animals, including all domestic animals, have blood corpuscles of a mean diameter, less than 1-3500 of an inch, and, in fact, those animals which, as a rule, are blamed for blood stains found on the clothing and apparel of criminals (ox, pig, horse, sheep and goat), have corpuscles with an average diameter less than 1-4000 of an inch (while all birds and fishes have oval corpuscles); and for a microscopist to say that such blood might be confounded with human, is preposterous and ridiculous under the present state of knowledge, especially if the question relates to fresh or unaltered blood.

It must also be remembered that with but a few exceptions, all the animals whose red-blood corpuscles approach in size those of man are inhabitants of either the tropics (South America, Australia or Africa) or the Arctic regions, unless found caged in a menagerie. The exceptions are such animals as the guinea-pig and opossum, the geographical distribution of which is also quite limited. Therefore a suggestion of the blood-expert that any one of those animals was to be blamed for the blood stains upon a person accused of murder, would be met with ridicule by the court, jury and public ; in fact, it is out of place for the expert to make any suggestions at all of that kind. (See section on expert testimony.)

The suggestion that human blood may be mistaken for ox blood, or the blood of any domestic animal, on account of variations in blood corpuscles, is also out of place, since such variations rarely amount to more than one to three per cent., and conclusions as to the kind of blood are not drawn from the measurements of a few of the largest or smallest corpuscles. Conclusions are deducted from the mean of hundreds of measurements of average sized, round, well-shaped corpuscles, of which there are at least ninety per cent. in good preparation and under favorable conditions.

It is a difficult task sometimes to diagnose rabbit's and dog's blood from human blood; the average diameter of the corpuscles of these animals being about 1-3600 in.; but only under unfavorable conditions. Fresh or well-preserved blood of these animals can be easily distinguished from human blood, by the quite appreciable smaller diameter plainly seen under high amplification. When it comes to diagnose guinea-pig's blood from that of man, then, however, I would hesitate to make a positive distinction, since the difference in diameter between the two is too insignificant.

*Conclusions Regarding Examination of Fresh Blood.*

1. The blood corpuscles of birds, fishes and reptiles being oval and nucleated can never be mistaken for human blood.

2. Fresh human blood cannot be mistaken, under the microscope, for the blood of any animal, the corpuscles of which have a mean diameter of less than 1-4000 or even 1-3600 of an inch.

3. (*a.*)—If the average diameter of blood corpuscles in fresh blood is less than 1-4000, then it cannot possibly be human blood.

(*b.*) If the diameter is more than 1-3500, then it may be human blood.

(*c.*) If the blood corpuscles, after exhaustive measurement, give a mean diameter of more than 1-3300, then it *is* human blood (provided it is not the blood of one of the wild beasts referred to).

So far we have considered exclusively fresh blood, and the conclusions stated above referred to the examination of fresh blood, or blood well preserved.

We will now enter upon the consideration of a subject more difficult, viz.: the *diagnosis of human blood, in a dried state, in criminal cases.*

III. Diagnosis of Human Blood in a Dried State and Blood Stains in Criminal Cases.—On rare occasions, liquid, or freshly clotted blood at a scene of murder, is to be examined, and its source to be established by the medical expert.

The procedure of examination is the same as that described in the former section for fresh blood.

*PLATE V.*

FIG. 13. HUMAN BLOOD CORPUSCLES RECOVERED FROM DRIED CLOT, TWO DAYS OLD. Magnified 2250 Diameters.

FIG. 14. OX BLOOD. Fresh Dry Preparation. Magnified 2250 Diameters. Both Photographed Under Same Amplification, 1-18 Zeiss Hom Immersion and Projection Eye-Piece.

It is impossible to distinguish arterial from venous blood, yet, if the blood is sprinkled over a considerable area, then it is likely to be the former, whereas venous blood is likely to be in larger quantity in a mass and covering less space. Venous blood is just as red as arterial, when exposed to the air.

Sometimes the question arises, whether blood is menstrual, and very frequently, whether its source was from the bleeding of the nose. Occasionally, blood specks upon linen garments are attributed to the biting of insects. Microscopical examination shows menstrual blood to contain a great deal of mucus and vaginal (large, elongated, flat) and uterine (columnar, ciliated) epithelial cells; the red blood corpuscles do not form rouleaux, and when fresh the blood has an acid reaction and is not coagulable.

Blood from the nose (epistaxis) also contains a large amount of mucus, may contain large, columnar, ciliated epithelium from the snyderian membrane. The blood of epistaxis may contain much fibrine and may be coagulable; reaction neutral. The elongated shape of the blood stains, and the location, direction and relation of the stains to each other, may be diagnostic.

Blood stains, due to insect bites, are more or less peculiar in their location and distribution, and it is well to trace their source to the wearer of the garments. The blood stains from insect bites, etc., coming from the body of the person are more prominent on the inside of the fabric of the garment he wears. It is important in all cases of blood stains upon garments to examine the person of the accused.

The *age of dried blood stains* is usually impossible to establish, because blood, once well dried, does not undergo any alterations; yet a freshly dried clot will dissolve much more readily in water than an old one, whereas, dried blood, a day or two old, will disintegrate and will show liberated blood corpuscles often immediately from the effect of proper reagents. The facility of the disintegration is more difficult in direct proportion to the age of the clot (see table of experiments). This, however, depends much upon the conditions under which the blood was kept, and I know it to be impossible to tell whether a blood stain is ten days or five years old. There is said to be a possibility of fixing the age of blood stains from the condition of the coloring matter.

In one instance a hardened drop of blood upon a garment of a supposed murderer proved to be more or less liquid in its interior after the lapse of two days. This disproved his claim that the blood stain was three weeks old.

Well dried and preserved blood and blood stains and likewise mounted specimens of blood for microscopic demonstration keep indefinitely.

*Is it Human Blood ?* In some cases the expert is required merely to establish the presence of blood without regard to its source, this being often established by witness testimony; usually, however, he is required to tell whether it is human, or blood from some other animal.

In former days great stress was laid upon the smell of the blood as developed upon the addition of sulphuric acid (1 part of blood to 1½ parts of sulphuric acid) and application of heat. This test was introduced by Burruel[1] in 1829, when a case of murder was decided upon the evidence derived from this test, the celebrated Orfila coinciding with him. Subsequently the sulphuric acid test was adopted all over Europe, and many cases of murder were decided by it. Burruel claimed that an odor was developed by this test peculiar to the animal from which the blood was taken (blood of horse—stable smell; cow—cow stable smell; dog—dog smell; but particularly, Burruel claimed, do peculiar odors develop in the case of cat, sheep and goat). Ritter[6] (1846), in an elaborate essay, for which he received a prize from the German government, proved that blood can be diagnosed in criminal cases with great certainty by combining the sulphuric acid test with the measurement of red corpuscles. It appears that he was the first to claim reliable results from the micrometry of blood in criminal cases.

For the physical properties of dried blood, as well as for the consideration of the conditions which produce various changes, see I. and II. Sections.

Dried blood presents itself to the examiner in various forms, either in dried masses embedded into substances or in the form of stains upon various substances, such as wood, stone, glass, instruments, and missiles of various kinds, and various kinds of apparel, such as cloths, linen, leather, etc. *Blood stains are best seen by artificial light.*

The examiner must note with precision the general appearance (whether in spots, smears or drops), the exact size, shape, number, location and distribution of blood stains upon the articles submitted to him. He must determine whether water was applied, as in washing. He must also carefully note any extraneous substances associated with the blood, such as fragments of mineral substances, wood, wool and other fabric, bone, hairs, spermatozoa, epithelial, muscular and any other animal and vegetable cells. (See Plate VI.) The presence of any such substances in the blood may sometimes be indicative of its source, and, as well as the surroundings of the case, should be taken into consideration and may aid in the decision, whether the blood is human or not. The court always sustains any point of the expert that is properly demonstrated.

The examination of dried blood *en masse* gives better results than mere stains, the shape of the corpuscles being better preserved in the former than in the latter. As to stains proper, those upon any substance which does not absorb the blood give better results; viz., stains upon glass, stone, metal, etc., are better for examination than upon soft wood, and better upon cloth than upon linen. For examination a granule of dried blood, no matter how small, is preferable to any even larger diffused stain on a fabric.

In order to examine blood in this form, the corpuscles which are glued together in a mass or are adherent to the substance upon which found, have to be freed from it and each other by macerating in certain liquids, and brought back to their natural shape.

*The liquids employed for remoistening and disintegrating dried clots* must be of such nature as to produce the desired effect without doing harm to the corpuscles. If the question is simply to determine the presence of blood corpuscles, then almost any liquid may be employed, such as water, alcohol, oil, glycerine; but in order to preserve or restore the shape of the corpuscles, various liquids have been suggested, which will be enumerated further on.

PLATE VI.

Fragments of various substances that occasionally may be seen in microscopical preparations and in blood stains: (*a*, and *b*.), wool; (*c*.), cotton; (*d*.), linen; (*e*.), silk; (*f*.), feather; (*g*.), mycelium and spores of a mould fungus; (*h*.), mucus; (*i*. and *j*.), rolled up masses of bacteria and granular debris; (*k*.), human hair: (*l*.), cork; (*m*.), vaginal epithelial cell: (*n*.), starch; (*o*.), wood splinter. (Magnified 300 diameters.)

In nature, these liquids are of two kinds: First, such as have the property of dissolving the fibrine which glues the corpuscles together; and, secondly, such liquids which will restore and preserve the shape of the corpuscles.

The best liquid for this purpose is a strong (30 per cent.) watery solution of caustic potash, introduced for examination of blood stains by Brücke and Virchow, in 1854, which usually fulfills both requirements, though not always.

Another good liquid for remoistering blood is Müller's fluid, first suggested by Prof. Rudnew, of St. Petersburg. This liquid, I prefer it when mixed with a little (5 per cent.) of glycerine and then diluted by water to the same specific gravity as blood serum (1028), often gives remarkable results. The composition of these liquids, as well as that of some others that may be applied in the remoistering of blood, are as follows:

1. *Virchow or Moleschott's Liquid.*

| | |
|---|---|
| Caustic Potash | 30 to 33 parts. |
| Water | 70 " |

2. *Muller's Fluid.*

| | |
|---|---|
| Bi-chromate of Potassium | 2 |
| Sulphate of Sodium | 1 |
| Water | 100 |

3. *Wilbert's Fluid.*

| | |
|---|---|
| Bichloride of Mercury | 0.5 |
| Chloride of Sodium | 2.0 |
| Water | 100 |

4. *Pacini's Liquid.*

| | |
|---|---|
| Water | 300 |
| Glycerine | 100 |
| Chloride of Sodium | 2 |
| Bichloride of Mercury | 1 |

5. *Ranvier's Liquid.* (Iodized Serum.)

| | |
|---|---|
| Potassium Iodide | 2 |
| Iodine, sufficient for saturation | |
| Water | 100 |

6. *Malassez Artificial Serum.*

Solution of Gum Arabic, sp., gr. 1020.
Solution of Chloride of Sodium, sp., gr. 1020.
Solution of Sulphate of Sodium, sp., gr. 1020.
Of each equal parts.

7. *Roussin's Liquid.*

| | |
|---|---|
| Glycerine | 3 |
| Sulphuric Acid | 1 |
| Water sufficient to make the liquid of specific gravity | 1028. |

8. *Robin's Solution* is a saturated solution of sulphate of sodium.

9. *Richardson's Salt Solution.*

| | |
|---|---|
| Chloride of Sodium | 0.75 |
| Water | 100. |

Having the corpuscles isolated by this liquid, he stains them with a little aniline or iodine.

10. *Welcker's Fluid.*

| | |
|---|---|
| Glycerine | 1 |
| Water | 7 |

11. He also used the following solution (artificial serum):

| | |
|---|---|
| Chloride of Sodium | 4 |
| Egg Albumen | 300 |
| Water | 2700 |

12. *Malinin's Solution.* Saturated alcoholic solution of caustic potash (90 per cent, alcohol).

Either of these solutions may be used, the author of each claims for his own the best results. It might be well in an important investigation to experiment with several solutions, because sometimes one, sometimes another, yields better results. Proper manipulation and some experience in this kind of work are, however, more essential factors in successful preparations than any particular liquid employed.

I stated above that Müller's fluid and very strong solutions of caustic potash are the two reagents which in my hands gave the best results. In order to obtain the largest possible quantity of unaltered measurable corpuscles from old dried clots and blood stains, I found that the application of slight heat for several days and of moisture (to prevent evaporation) to be of advantage. The procedure I adopted is as follows :—A small granule of the suspected blood or a fibre from the blood-stained fabric is placed on a glass slide in a drop of a 30 to 35 per cent. solution of caustic potash and covered with a cover glass. If the blood stain was recent, the disintegration of the clot commences at once, and the isolated corpuscles separate and swim swiftly through the liquid if the stage of the microscope is slightly inclined. It is quite interesting to observe how perfectly well-shaped blood discs will tear themselves away from the original formless brown mass.

In direct proportion to the age of the stain, from one to within ten days, the softening of the microscopic blood mass and the isolation of the corpuscles is protracted. In dried blood older than ten days the ratio of softening or disintegration cannot be well observed, and a stain of two years old behaved like one of ten days.

The examination can be made under comparatively low amplification, such as 300 to 500 diameters; but when measurements are necessary, then an immersion lense, giving a magnifying power of about 1,000 diameters, better be substituted.

Sometimes but a few well-shaped measurable corpuscles are seen, but quite often, in successful preparations from recent blood stains, nine-tenths of the corpuscles in a certain microscopical field will appear quite perfect and fit for measurement.

If the blood specimen is slow in disintegrating and the corpuscles imperfect in appearance, then I adopt the following procedure :—The glass cover beneath which the blood fragment is mascerating on the reagent is ringed with a little oil, or, still better, with some cement, in order to fasten it and to prevent evaporation, and placed in a moist chamber (a glass vessel, lined with moist paper and covered). The chamber itself put in a water-oven (incubator such as used in Bacteria investigation) and subjected to uniform slight heating, not exceeding 100° F., and kept there from one to three days, or as long as is necessary to obtain the desired result, the specimen being examined from time to time.

Care must be taken not to over-heat the preparation and guard against evaporation of contents, *i.e.*, of the liquid between the glass slide and glass cover in which the blood specimen mascerates. A number of experiments may be made simultaneously, some of the blood specimens being treated with a strong solution of caustic potash, others by Müller's fluid, the latter often succeeding in very old blood clots to restore shrivelled and to isolate perfect corpuscles when the former fails.

Whereas, the Müller's fluid with glycerine must be diluted with water in order to obtain the desired specific gravity, a peculiarity in the action of the caustic potash solution must be borne in mind, viz. : that the stronger the solution the better its effect, whereas, weak solutions of this reagent (caustic potash) destroy the blood corpuscles or reduce them in size by making them spherical. A strong solution (30 to 35 per cent.) gives most beautiful results: The red blood corpuscles have an absolutely natural appearance; retain their perfect color and shape or sometimes resume it, if previously lost, form rouleaux, show the normal bi-concavity of the discs, and even show normal diameters on measurement; in short, behave like normal blood. Such is the case when the blood stain was a recent one, and, in fact, the rapid appearance of such good and perfect pictures under the microscope are indications of the recency of the blood stain.

FIG. 15.

Suspected blood in a criminal case, from minute blood clot two days old, caustic potash preparation × 830 diam. Micrometry showed 1-3185 in. as the average diameter of the corpuscles.

All blood stains only a few days old that I ever examined in criminal cases, as well as those produced experimentally, behaved in the manner described. (See Fig. 15.)

When the dried blood or blood stain is more than a week or ten days old, then the reaction is less prompt, much fewer corpuscles are perfect and measurable, but still enough to make the result of examination quite satisfactory.

The copy of my photo-micrograph, Plate V, Fig. 13, represents red blood corpuscles that I restored from a minute blood clot taken from within the seam of a handkerchief which was claimed by the defendant was stained by ox blood. Measurements showed the average diameter of one hundred of the largest corpuscles to be 1–3185 of an inch, which proved the range of human blood corpuscles. A similar preparation of ox blood corpuscles (Fig. 14) was simultaneously and under similar amplification photographed, and gave a micrometry of 1–4168 of an inch. The difference in size is also quite obvious in the photomicrographs 13 and 14.

In blood stains we meet quite often with a few or with the majority of the red-blood corpuscles shrivelled or contracted (crenated) from the effects of drying. (See also physical properties of blood, section I. of this article.) When such corpuscles are remoistened with liquids of less or of the same density as the blood serum (spec. gravity 1028) they only partially and very slowly regain their normal shape. More often, however, they become spherical, and consequently diminished in diameter. This is even the case with the oval oviparous blood which we then are able to tell from mammalian only by the size and by the presence of the nuclei. But we further observe that under equal conditions there is a certain definite ratio in the diminution in size of these artificially spherical corpuscles which is the same in all the various animals. It can be easily observed that human corpuscles thus altered appear of the size of the corpuscles of the ox, and that similarly spherical ox blood corpuscles appear reduced to about the diameter of sheep's corpuscles. It was thus necessary to establish what the average sizes of these spherical corpuscles were, whether the ratio of diminution was really uniform in all animals, whether it was constant, and, per consequence, whether it could be applied and relied upon in the diagnosis of any blood thus altered.

It was evident that in the micrometry of blood corpuscles in blood stains at least two scales of measurements* for each animal must be established. Further, that the distinction between normal disc-like, bi-concave corpuscles (the larger ones) and corpuscles that had become artificially spherical (and hence the smaller ones) must be rigorously observed in the micrometry of prepared blood of every animal examined. Finally, it was obvious that only strictly disc-like and fully spherical corpuscles should be submitted to measurements, and that any transitional stages in the corpuscles should be carefully avoided.

Although the best occasions for experiments were furnished me in the ample material from actual criminal cases (the source of the blood being subsequently confirmed by either confessions of the criminals or by witness testimony), I made invariably in connection with every case some control experiments and measurements upon blood prepared under known conditions. The combined average results of some of these measurements made by myself, and which bear directly upon these questions, I present in tabular form.†

The following table of experiments and measurements explains itself : (See page 295.)

Conclusions Regarding Diagnosis of Blood in Its Dried State and in Blood Stains.—We have seen that blood can be diagnosed in its dried state and in blood stains with the same certainty as fresh blood, provided the drying of the blood was rapid and perfect. The blood corpuscles preserve fully their color, size, shape (bi-concavity) and even their arrangement into rouleaux (only occasionally are such corpuscles a trifle smaller than in fresh blood). But no diagnosis should be made unless the shape of the corpuscle is well taken into consideration, all abnormally small and disfigured corpuscles excluded. At least 500

---

* Masson, however, thinks that the "crenated" corpuscles can be more relied upon for uniformity in size than the spherical ones, a proposition with which I do not agree.

† Being limited in space for this article, I must, for the present, omit all less essential details and a plainer classification of the experiment.

### COLLECTIVE RESULTS OF SOME OF THE SERIES OF MEASUREMENTS OF RED BLOOD CORPUSCLES IN BLOOD STAINS AND IN EXPERIMENTALLY DRIED BLOOD.

Normally shaped (bi-concave, disc-like) corpuscles only being measured.

| Source of Blood. | Upon what Substance. | Age of Stain. | Condition, or how Prepared. | Number of Individuals Examined. | Number of Preparations Made. | Reagents used for Remoistening. | Time of effect of Reagents. | Per cent. of Measurable Corpuscles in each preparation. | Total Number of Corpuscles measured. | Average Diameter in inch. | Normal Diameter of Fresh Blood. |
|---|---|---|---|---|---|---|---|---|---|---|---|
| Man. ........ | Knife and Glass. | 2 days. | Rapidly dried ................ | 10 | 30 | *K. O. H. | 5 to 30 min'ts. | 20 to 50 | 1000 | 1-3260 | 1-3200 |
| Man. ........ | Cloth .. . . ..... | 7 days. | Slowly dried ................ | 2 | 10 | K. O. H. | ½ hour to 2 dys | 5 to 20 | 250 | 1-3300 | 1-3200 |
| Man. ........ | Wood and Linen. | 10 days. | Slowly dried ................ | 4 | 20 | *M. F. | 2 hrs to 2 dys. | 5 to 15 | 200 | 1-3300 | 1-3200 |
| Man. ........ | Paper . ............ | 14 days. | Decomposed from moisture. | 1 | 10 | M. F. | 3 days. | not me | asurabl | e. | |
| Man. ........ | Knife. ... ...... | 2 years. | Well dry preserved......... | 1 | 10 | K. O. H. & M. F. | 2 days. | 10 to 50 | 400 | 1-3240 | 1-3200 |
| Man. ........ | Stone............. | 6 years. | Well preserved. ............ | 1 | 30 | K. O. H. & M. F. | 3 days. | 5 to 20 | 500 | 1-3320 | 1-1320 |
| Guinea-pig... | Glass............. | 7 days. | Rapidly dried stains......... | 6 | 18 | K. O. H. & M. F. | 1 to 2 days. | 10 to 40 | 500 | 1-3460 | 1-3400 |
| Wolf ........ | Glass............. | 7 days. | Rapidly dried stains..... ... | 1 | 50 | K. O. H. & M. F. | 1 to 2 days. | 5 to 20 | 1000 | 1-3450 | 1-3450 |
| Dog. ....... | Cloth............. | 7 days. | Rapidly dried stains......... | 4 | 12 | K. O. H. & M. F. | 1 to 2 days. | 5 to 50 | 500 | 1-3650 | 1-3580 |
| Rabbit ...... | Knife......... ... | 7 days. | Rapidly dried stains......... | 10 | 30 | K. O. H. & M. F. | 1 to 2 days. | 5 to 50 | 1000 | 1-3700 | 1-3662 |
| Ox. ......... | Cloth............. | 7 days. | Rapidly dried stains......... | 10 | 30 | K. O. H. & M. F. | 1 to 2 days. | 20 to 40 | 1000 | 1-4240 | 1-4200 |
| Sheep. ...... | Glass............. | 7 days. | Rapidly dried stains......... | 3 | 9 | K. O. H. & M. F. | 1 to 2 days. | 50 | 500 | 1-5060 | 1-5000 |
| Goat......... | Knife..... ....... | 7 days. | Rapidly dried stains......... | 3 | 9 | K. O. H. & M. F. | 1 to 2 days. | 50 | 500 | 1-6200 | 1-6100 |

* "K. O. H." stands for 33 per cent. Solution of Caustic Potash. "M. F." for Muller's fluid.

measurement should be made in establishing the average diameter.

It is also seen from these experiments that, in some instances, no reliable measurements could be made at all.

Blood corpuscles once entirely deformed from decomposition before drying, cannot be restored to their normal shape by the employment of any known reagent.

Blood which had been subjected to the slow effects of moisture before being dried, but without any putrefactive change having occurred, is also unsatisfactory for examination and diagnosis as to the source of blood. Yet such specimens are rare, and occasional negative results in the diagnosis of blood stains should by no means throw doubt upon the reliability of results in other cases, the diagnosis being possible in 90 per cent.

All rules regarding diagnosis of fresh blood (see Section II.) hold good in the diagnosis of remoistened blood stains in criminal cases.

If a great part of the corpuscles are found distorted and their shape is not easily or not at all restored by remoistening, then we can conclude that the blood stains have been washed or soaked and subsequently dried.

The diagnosis should not be declared impossible as long as there are some perfect bi-concave corpuscles present, even if the bulk of the corpuscles are distorted, for we have seen that even altered corpuscles can be measured.

If the corpuscles are spheroidal from absorption of moisture or crenated from drying, they may still be diagnosed, because such changes as seen from the tables are the same in the corpuscles of all animals and have really their proportionate and corresponding ratio of alteration in form and diminution in size, the range or scale of diminution being always alike in the same animal.

The red-blood corpuscles that have become spherical from imbibition of liquid have thus presented in my experiments the following average diameters in the various animals :

1. Man, 1–4300 in.,
2. Guinea-pig, 1–4500 in.,
3. Wolf, 1–4600 in.,
4. Dog, 1–4800 in.,
5. Rabbit, 1–4900 in.,
6. Ox, 1–5600 in.,
7. Sheep, 1–6700 in.,
8. Goat, 1–8100 in.

These figures show that the diameter of the artificially spherical corpuscles in each animal is just about one-third ($\frac{1}{3}$) less than that of the normal bi-concave or disc-like corpuscles of the same animals :

1. 1–3200 in. (Man) reduced $\frac{1}{3}$ = .–4267 in.

2. 1–3400 in. (Guinea-pig) reduced $\frac{1}{3}$ = 1-4533 in.

3. 1–3450 in. (Wolf) reduced $\frac{1}{3}$ = 1–4580 in.

4. 1–3580 in. (Dog) reduced $\frac{1}{3}$ = 1–4773 in.

5. 1–3662 in. (Rabbit) reduced $\frac{1}{3}$ = 1–4882 in.

6. 1–4200 in. (Ox) reduced $\frac{1}{3}$ = 1–5600 in.

7. 1–5000 in. (Sheep) reduced $\frac{1}{3}$ = 1–6667 in.

8. 1–6100 in. (Goat) reduced $\frac{1}{3}$ = 1–8133 in.

The close similarity between the figures of my micrometry of the corpuscles and the figures of the right column is apparent ; the latter being the accurate scale.

All kinds of blood behaving alike as regards drying, shrinkage, swelling, etc., and, knowing the conditions under which these changes take place to be alike in all, these alterations should not necessarily debar us from making a diagnosis. The examiner must, however, express himself guardedly about such results.

Corpuscles never increase perceptibly in size, and if they diminish in diameter they change from the typical bi-concave shape to the spherical ; *e.g.*, a human corpuscle which has become spherical and approaches the size of the blood corpuscle of the ox, can readily be distinguished from it by not being bi-concave like the normally shaped ox blood corpuscle. But if the corpuscle is disc-like and too small, then it cannot be human.

Unless the examiner has some experience in microscopy, hæmatology, and a proper perception of sizes and shapes, he must postpone diagnosis of blood corpuscles in criminal cases until he has acquired such experience.

### Errors Regarding Suspected Blood-Stains.

1. *Various dyes or paints* may produce spots which resemble blood-stains. These are easily determined by the microscope: *No blood-corpuscles—no blood-stain.* Chemical tests may, however, be tried in addition.

2. A *Bacterium*, the micrococcus prodigiosus, gives rise sometimes to beautiful blood-red spots by vegetating in large colonies upon damp tones and other substances; even upon old moist garments that have not been disturbed for some time. I meet it occasionally upon the skin and apparel of bodies disinterred some months after death in medico-legal cases. This microccocus is, however, much smaller than red-blood corpuscles.

3. *Spores of various fungi* which closely approach red-blood corpuscles in size and color are a more dangerous source of error, met in various stains as they often are. They are, however, never disc-shaped, are invariably more or less oval, occur often in pairs and may have buds upon them. Moreover, the adult mycelial threads are usually seen in connection with the spores. Upon plate VI. I have pictured (besides fragments of various substances that may be met with in blood-stains) at figure "G" such a mycelium of the pennicillium glaucum and some free spores of the same.

4. The spheroidal, small, dark yellow crystals of urate of ammonia, I know of having been mistaken for red-blood corpuscles; but they are larger than the latter, uneven in size and often spiculated.

5. *Minute oil drops* are also a dangerous and frequent source of error. The only conspicuous distinction between oil drops and red-blood corpuscles that have become spherical (as they always do when macerating in a liquid of low specific gravity) is the marked unevenness in size in the former. (These sources of errors, with exception of spores, are not mentioned in text-books of Medical Jurisprudence—whereas, a lot of other things that have not the least resemblance with red-blood corpuscles *are.*)

IV. Expert Testimony upon Blood in Criminal Cases.—There should be no discrepancy as regards facts established by scientific, accurate observations and experiments.

The dignity of science should not be molested by improper legal consideration, neither on the part of the prosecution nor on the part of the defence of criminals, such as the case may present. The truth is, however, often obscured by submitting to the expert only such questions that may further the interest of one side only, or by unduly influencing the jury by asking the expert misleading questions, especially if he is not permitted to qualify his answers and the counsel on the opposite side is taken unawares. Yet these are questions which simply concern the law and its expounders.

On the other hand, the medical witness or expert may himself do a great deal of harm to justice if he is careless in his expressions or makes overstatements.

There are striking instances of peculiar ideas regarding expert testimony in blood investigations.

Prof. Wormley,[46] whose article gives evidence of a great deal of personal, reliable work, and thorough familiarity with the literature of the subject, after expounding in the clearest manner the accuracy and reliability of microscopical research in regard to the diagnosis of blood, draws rather peculiar conclusions. Scientifically, they are correct, but practically, they are not applicable and not just, since they might be used in the unscrupulous defense of real criminals with the object of obscuring the truth before a jury. Prof. Wormley, who lays great stress upon the difference in the size of corpuscles, records only the guinea-pig and some wild animals, such as the capybara, seal, beaver, musk-rat and opossum, as having corpuscles approaching in diameter the size of that of man. He thus evidently implies that blood corpuscles having the size of 1–3250th of an inch are human if they do not pertain to any one of the animals enumerated ; but the question as to whether one of these wild beasts comes into consideration, the jury will decide and not the expert.

It does not follow that because there exist certain wild animals which have blood corpuscles of the same diameter as man, that the expert must refrain from expressing a definite opinion as to a certain specimen of blood being human, if he finds that it corresponds to human blood, and the evidence given does not show any one of the animals in question were within reach at the time.

The following passages from the article of Prof. Wormley, referred to, may be of use to the reader interested in the defense in criminal cases, and hence I quote them in full :

"This difficulty of individualization arises from the fact, as we have already seen, that the average diameters of the corpuscles of the different mammals are in many instances at least practically the same, and these averages, for the most part, pass by imperceptible gradations throughout the entire class. Thus, virtually of the same size as the corpuscles of man, are at least those of the guinea-pig, musk-rat, seal, beaver, opossum and capybara, whilst those of certain other animals are but slightly larger and might be reduced in size to those of man."

"Hence, then, *the microscope may enable us to determine with great certainty that a blood is not that of a certain animal and is consistent with the blood of man; but in no instance does it, in itself, enable us to say that the blood is really human, or indicate from what particular species of animal it was derived.*"

"There seems to be much misunderstanding as to the true value of this instrument in investigations of this kind (?) it being regarded by some as nearly or altogether useless for this purpose, whilst others claim for it results wholly at variance with the facts in the case. This, like many other tests, has its fallacies, and if these, in a given case, cannot be reasonably met, the accused should have the benefit of the doubt."

WOODWARD,[34 and 35] a prominent microscopist, argues in the same strain, but he goes much farther than Prof. Wormley, and denounces the measurements of blood corpuscles altogether as wholly unreliable for establishing a diagnosis between human blood and that of *any* animal! That his own (Woodward's) measurements (which were limited to the corpuscles of man, dog and guinea-pig) were all erroneous, I have shown above (see page 269). When Woodward further says that "it is not rare to find specimens of dogs' blood in which the corpuscles range so large that their average size is larger than that of many samples of human blood," then it is quite evident that his studies were made upon blood under unequal physical conditions and varying amplification, and that his deductions from such observations (which are contrary to the experience of every hæmatologist) were quite erroneous and misleading. Yet he concludes his paper by saying: "It is sufficient to demonstrate the reckless temerity of those who would attempt to discriminate human blood from that of animals."

In another article Dr. Woodward gives the following gratuitous advice:

"In conclusion, then, if the microscopist, summoned as a scientific expert to examine a suspected blood stain, should succeed in soaking out the corpuscles in such a way as to enable him to recognize them to be circular discs, and to measure them, and should he then find their diameter comes within the limits possible for human blood, his duty, in the present state of our knowledge, is clear. He must, of course, in his evidence, present the facts as actually observed, but it is not justifiable for him to stop here. He has no right to conclude his testimony without making it clearly understood, by both judge and jury, that the blood from the dog and several other animals would give stains possessing the same properties, and that neither by the microscope nor by other means yet known to science, can the expert determine that a given stain is composed of human blood, and could not have been derived from any other source. This course is imperatively demanded of him by common honesty, without which scientific experts may become more dangerous to society than the very criminals they are called upon to convict."

Besides being uncalled for and misleading to make such suggestions, it is wrong to do so.

The burden of proof to account for the kind of blood in any given blood stain rests upon the defendant and not upon the expert.

Looseness in statements may interfere with the conviction of the real criminal; on the other hand, it must be remembered that accurate statements may save an innocent man; for instance, when the expert proves, contrary to all assertions, that a certain blood is really not human.

Undecided testimony in either case under circumstances where positive evidence can be given, is a calamity to justice.

I beg leave to relate the following peculiar case from my own experience:

On the 17th of March, 1867, a murder was committed in one of the principal towns of the Balkan States, which gave me the first opportunity of examining blood stains in a medico-legal inquiry, the question being to establish whether the blood corpuscles found on certain objects were those of a man or those of a bird.

When I was compelled to testify in court I swore that the blood was mammalian, and not that of a bird, but I was finally forced to withdraw my opinion. I was much intimidated by the arrogance of the experts for the defense, and the brilliant array of counsel.

It was maintained that the drying of the blood produced such a change in the oval corpuscles as to make them indistinguishable from the round mammalian blood discs; opinions of scientists to that effect being quoted from text books. This influenced the jury in favor of the defendant.

The circumstances of the case were as follows:

A young and very rich nobleman, or rather boy, because his age was little over twenty, while on a debauch, killed a coachman while driving out in a sleigh. The lifeless body was found with skull crushed on the road a few miles from town. The young man having returned with his sleigh, disclaimed any knowledge as to the whereabouts of his driver.

The only evidence that tended to bring him into connection with the crime were large blood spots upon his shirt sleeve, upon his clothing and upon the sleigh, which he plausibly explained as having been produced by his carrying recently killed birds, he being a passionate sportsman. On the trial he was acquitted.

Five years later, in May, 1872, the father of that same young man was found dead in his private residence with several bullet holes in his head, one of the bullets having entered the back of the neck, another posterior to the right ear, and a third one passing across the mouth through both cheeks.

The victim's mouth was full of blood and blood had spurted upon the furniture and floor.

It was indisputably in evidence that the son (the same young man acquitted of the murder of the coachman) was his father's murderer.

Large blood stains were plainly seen upon his clothing, and he was the last person who had seen the victim alive. There was also sufficient motive: the inheritance of a large fortune, to the exclusion of several sisters and a number of other relatives. He had been irresponsibly drunk for some time, leading a most dissipated life, and in one of his debauches had confessed the murder of his father. He was arrested, the case closely investigated and put on trial. Yet the expenditure of enormous sums of money and the strange disappearance of all the important witnesses for the prosecution, and again the inability to determine the character of the blood stain, saved him.

This question of blood stains again came up prominently at this trial. I again participated in the microscopic part of the investigation, and was urged by the Court to declare the stains to be those caused by human blood, and not wolf's blood as was claimed by defendant (the hunting of wolves in that country being an exceedingly common sport in certain seasons, and the defendant claiming and bringing witnesses to prove that he, shortly before the murder, participated in that sport). Again high paid experts and counsel for the defense guarded the interests of the murderer at the trial.

Yet if the difficulty existed at the first trial, where I had failed to impress the jury with the difference between bird blood and human blood, how hopeless was it now, when the question concerned two mammalian bloods, so closely resembling each other in size of corpuscles. I spent much time in the preparation of the case, and made numerous experiments in connection with the investigation, was aided by Prof. Rudnew of St. Petersburg, and I had visited Berlin to consult Prof. Virchow. The latter, however, utterly discouraged me, saying that no one is justified in putting the question of a man's life upon the uncertain measurements of dried blood corpuscles. The defendant, as already mentioned, was again acquitted. He subsequently squandered his father's large fortune, made his mother and three sisters and their families paupers, and finally shot himself, not, however, without leaving a confession that he was the murderer of his father and his coachman. Rumor had it that he had also killed a pawnbroker shortly before his suicide.

I have since given considerable thought to the results and consequences of these examinations of blood stains, and to my testimony in these two trials, particularly the first one.

The reason why the murderer was not convicted upon the first trial, was solely from the fact that the prosecution did not prove that the blood stains could not have been bird's blood.

It was certainly a case in which a conviction could have been obtained if I had stood up for the cause of science and insisted that the blood was not from a bird, but was of mammalian origin.

It is unknown in the annals of criminal cases that an innocent man has ever been convicted by expert testimony upon blood stains; while, on the other hand, in perfectly clear cases of murder, where there was no doubt whatever expressed by any one as to the source of the blood, the perpetrator of the crime went free, on account of the vacillation of the experts.

Another case in which the jury decided chiefly upon the result of microscopical examination of blood stains was as follows:

On the first of February, of the present year, I was called upon to discriminate again before the court of a neighboring State between human blood and what was claimed to be the blood of a bird.

The question being to decide between human blood and chicken blood, I declared the stains to be due to human blood.

This testimony being the direct connecting link between the murder and the defendant, he was convicted and was executed. Previous to execution he confessed the murder.

In the time that elapsed betwen the two examinations of blood stains, in 1867 and 1888, I have made quite a number of examinations of alleged blood stains in this and other States.

My examinations have not always led to a positive determination of human blood. I have had some examinations turning out favorably for the defendants, while others resulted adversely to them.

Again, I have had cases in which I refused to testify as regards the kind of blood in given stains on account of the complete disintegration of the material offered, such as explained in the preceding section.

There are thus, from a legal standpoint, two classes of blood stains—*first*, those which we can determine; and *second*, those which we cannot; the former of which, however, occur far more frequently than the latter.

*The method of testifying in court*, as an expert upon blood examination, has more than once been the subject of consideration at the meetings of scientific bodies, and opinions have been expressed by medical jurists of high standing. I refer in particular to the question whether the fact already referred to, that there are certain animals which have blood corpuscles closely approaching in size those of man, should debar the medical expert from affirming that a certain blood is human. Further, whether it is proper for the expert, testifying as to the source of blood, to take into consideration the surroundings of the case in addition to the results of the microscopical examination; provided, of course, that he is also an expert in the question involved.

Although it is stated by such high authorities in medical jurisprudence as Caspar,[55] Taylor,[56] Tidy,[57] Fleming,[13] and others, "that the difference between the red corpuscles of man and other animals is too minute to render their positive discrimination possible and too insignificant to admit of its being used as the means of condemning a fellow creature to death," it must be remembered that these gentlemen did not measure any corpuscles themselves, and relied altogether upon certain authorities who denied the reliability of micrometry of blood corpuscles.

On the other hand, there are such high authorities in microscopy as Virchow,[11] Brücke,[10] Mandl,[4] and others, who, while fully acknowledging the reliability of the results of micrometry of fresh blood, deny our ability of diagnosing human blood in dry stains by this method; but it must be remembered that they have reference only to certain instances in the cases of dried blood, and that their writings on the subject date back as early as 1842 and 1857, and further, that it is to be hoped that they have changed their opinions by this time.

In the bibliography at the end of this paper, the reader will find a list of all the original observers, as far as I know, classified as to their views upon this subject, marked "positive" or "negative."

It is plain, however, that the great majority, if not all, of the recent observers in this domain (certainly all who have worked with improved instruments and employed lenses of high amplification and proper methods of micrometry) are in favor of judicious discrimination between human blood and that of animals.

In France a committee appointed by the Société de Médecine Légale,[20] composed of Monsieurs Mayet, Mialhe, Cornil and Lefort, decided that the expert measuring the corpuscles has the right to affirm whether or not they are human.

Other French Medico-Legal examiners, such as Lacour[48] and Masson,[47] who made most extensive researches upon blood stains, have testified as follows: "One can certify that corpuscles found in the blood under examination are in all points identical with those of man or of the guinea-pig, if they measure more than 1-127 millimetre.

The Russian Medico-Legal experts often testify directly that certain blood is human or not human. Professor Rudnew, of St. Petersburg, told me himself that he has testified in the affirmative in regard to human blood in blood stains. Dr. Malinan,[27] a prominent expert on blood in criminal cases, of Tiflis, Russia, makes the following statement: "If we find corpuscles in blood stains, the diameter of which is 0.0077 millimetres or more, then we can conclude that it is in all probability human blood," and he testified in Court to that effect.

Prof. Carl Schmidt,[7] of Dorpat, Russia, was also quite emphatic in declaring himself that certain blood is human blood if the corpuscles corresponded to the limits of certain measurements.

Dr. Hans Schmidt,[40] of Erlangen, Germany, who made a most excellent experimental investigation upon the subject of blood stains, and acquired a great reputation as an expert in his line, says in his monograph: "If the question is asked of the expert whether a certain spot is due to the blood of man or of a certain animal, he may answer the first part of the question in the affirmative under given conditions, while the second part of the question he cannot unconditionally answer."

The foremost authority on the discrimination of blood stains in this country is unquestionably the lately deceased J. G. Richardson,[23] whose writings on the subject have been translated in all the languages of the world, and I think that he never was surpassed by any one in the accuracy and reliability of his studies in the micromety of blood corpuscles. He concludes one of his excellent papers as follows:

"We are now able, by the aid of high powers of the microscope, under favorable circumstances, to positively distinguish stains produced by human blood from those caused by the blood of any of the animals enumerated (viz., the pig, ox, horse, sheep, goat and cat), and this even after the lapse of five years from the date of their primary production."

There are several other authorities who express themselves very positively about the mode of giving testimony (see Bibliography), the summary of which is certainly to the effect that the microscopical *expert* has the right to express himself quite definitely as regards the probable identity of blood. There is no living *expert* hæmatologist who at present would fail to express a more or less definite opinion as to the identity of blood if the condition of the specimen renders it possible; and further, if he does not wish to obscure the truth for either the benefit of the defence or that of the prosecution.

The exact expression to be used by the expert in testifying to the existence of human blood upon a certain substance under examination is not subjected to written laws or rules.

If the testimony is, as customary, worded—"*The blood is consistent with human blood*"—it is usually quite satisfactory to the prosecution, and is an expression sufficiently guarded.

If the question rests solely between human blood and ox blood, for instance, and the expert is asked, "*Which is it, if it is one of the two?*" then he can surely answer that "*the blood is identical with human blood,*" or, "*is human blood,*" if he found that the average diameter of the corpuscles corresponds to that of man, and not to that of the ox.

I do not think it necessary that the expert should be required to qualify his statement, regarding human blood, that the blood might be also that of a guinea-pig or musk-rat, or beaver or capybara, or that of any animal whose corpuscles approach in size to those of human blood. The Commonwealth need not consider such a question at all, because the burden of proof rests upon the defendant that he had come in contact with any one of those animals. To introduce such questions into the defense should not at all be permitted, unless there is some foundation for it. As a rule, however, the defendant always accounts for any blood stains upon his person or apparel. In fact, I have never met with one instance where this was not the case. I have never been asked the question regarding human blood unqualifiedly, but in all the cases the question to decide was whether the blood stains were due to the blood of man, or to that of a *certain* other animal.

It is perfectly proper to say, that "human blood cannot be told from the blood of *all* other animals." But from what we know about blood, it is very improper and misleading to the jury to put the question in this form, and to insist upon the answer to such a question by the expert to the exclusion of any further explanation. If, however, the question is put to the expert, "Can you distinguish human blood from that of all *domestic* animals?" the answer should be given in the affirmative, provided the guinea-pig is not considered as a domestic animal. I do not think that such answers are improper, especially when the question implies fresh or well-preserved blood.

*Conclusions regarding expert testimony.*

Under the present state of our knowledge upon and the means for blood examination, disputes about the kind or ource of blood should not occur, provided the specimen of blood under consideration is in such a state of preservation that the corpuscles can be measured at all. If the blood is well preserved, the expert can determine it easily; if, on the other hand, it is putrefied, he cannot.

The diagnosis, of course, should be limited to the identification of human blood, and the exclusion of certain given animals. If, for instance, a certain blood is not from the ox (which can be sworn to), then it is also not from the horse, pig, cat, etc., which have corpuscles nearly identical with the ox; though the blood *may be* derived, either from the sheep or goat, which are endowed with corpuscles still smaller, or from the dog, rabbit or rat, which have corpuscles larger than the ox, but smaller than those of man.

While these special differences are of no practical importance, because for the killing of a cat or a pig no one will be tried for murder, it is of great importance and quite convenient to remember that *all* domestic animals have corpuscles decidedly smaller than man, and that under similar conditions these relations are not disturbed by the drying of the blood in blood stains.

When the decision as to the source of blood in a criminal case is dependent upon or may be influenced by the testimony of the medical expert, the following requirements should be complied with regarding his testimony:

1. It must be proven that the blood causing the stains under consideration presented corpuscles fit for measurement, *i.e.*, was not putrefied.

2. The examiner must substantiate his testimony by microscopical slides prepared from the blood stains in question, and, if possible, present micro-photographs of the same.

3. He must have made not less than five hundred measurements of the corpuscles, and, if required, present a full statement of the series and methods of the measurements from which he drew his conclusions.

4. He must give full details of the methods he employed in the preparation of the blood stains under consideration, and demonstrate them before the jury, if required.

All disputes as to the reliability of the testimony should then be out of place.

In a few cases the opinion must be based upon a smaller number of measurements, and yet the result of the examination is, to say the least, equally reliable with other delicate tests, such as the various chemical tests for poisons, as, for example, the tests for arsenic when the latter is present in excessively small quantities. It is certainly equally justifiable to convict a man on the definite determination of a small number of corpuscles, as explained in the text, as to convict him on the presence of a small number of exceedingly microscopic crystals in a glass tube, which is done time and again. Indeed, the micrometry of blood is less liable to error than many chemical tests.

The expert should maintain the following precautions: He should see that the blood-stained articles are properly identified; he should receive them himself and not relinquish his possession of them until he testifies in court. He must guard against accidental stains, such as may occur from a careless packing together of the suspected articles with the clothing of the murdered person. He should examine them at once, and if a delay is unavoidable, they should be at once thoroughly dried to avoid decomposition of the blood.

If possible, it is well for the expert to inspect the scene of murder himself.

The present article is merely a short record and outline of the facts concerning blood examination with omission of details; it is largely made up from stenographic reports of my own lectures and demonstrations upon this subject. Hence it cannot pretend to be a success from a literary standpoint, or to be regarded a complete treatise upon this subject.

Yet I hope that this article will benefit those who wish to pursue active work in this line of study, or wish to read it for general information.

V.—BIBLIOGRAPHY.—*Comparative Studies upon Blood and Blood Stains.* Comprising all, or nearly all, the original articles on the subject to date.

Those observers who draw a definite distinction between human blood and that of the domestic animals, are marked "*positive;*" those undecided or adverse to this, are marked "*negative.*"

1. *Burrnel.* Mémoire sur l'existence d'un principle propre à caractériser le sang de l'homme et celui des diverse espèces d'animaux. Annales d'hygiene No. 6. Paris, 1829. (Positive.)

2. *Orfila.* Traité de Médicine Légale, 1829. 3 edit. T. II., p. 700. (Positive.)

3. *Bertazzi, G.* Mezzo per distinguere le macchie di sangue di diversi animali. Ann. univ. di med., Milano, 1839. (Positive.)

4. *Mandle, L.* Thèse de Paris, 1842. (Negative.)

5. *Nasse.* Wagner's Handwöterbuch der Physiology, 1842. (Negative.)

6. *Ritter, B.* Ueber die Ermittlung von Blutflecken an Mettallischen Instrumenten, Berlin, 1846. (Prize essay.) (Positive.)

7. *Schmidt, Carl.* Diagnostik verdächtiger Flecke in Criminalfällen ; Milan und Dorpat, 1848. (Positive.)

8. *Friedberg, H.* Forensische Diagnostic des Blutes, Berlin, 1852, Virchow's Archive, XII., 1857. (Negative.)

9. *Teichman,* Ueber die Krystallisation der chemischen Bestandtheile des Blutes. Henle's und Pfeiffer's Zeitsch. f. rat. Therapie T. III., p. 375. 1853. (Positive.)

10. *Brücke. C.* Ueber gerichtliche Untersuchungen von Blutflecken. Wiener Med. Wochenschrift, No. 23, 1857. (Negative.)

11. *Virchow, R.* Ueber die forensische Untersuchung von trockenen Blutflecken. Virchow's Archive, XII., 1857, p. 334. Also in his Gesam. Abhand., 1879, II., p. 548. (Negative.)

12. *Robin, C.* Mémoire concernment l'examen à l'aide du microscope de taches de sang, etc. Annales d'Hygiène et de Medicine Légale, 1857. T. VIII., p. 368, and 1858, X., p. 421, and 1859, XII., p. 150, (Positive.)

13. *Flemming, A.* Blood stains. Am. Jour. Med. Sciences, 1859, XXXV., p. 84–119. (Negative.)

14. *Pfaff, E. R.* Anleitung zur Vornahme gerichtärztlicher Blutuntersuchungen. Planen, 1860. (Positive.)

15. *Welcker, H.* Grösse, Zahl, Volumen Oberfläche und Farbe der Blutkörperchen beim Menschen und bei Thieren. Zeitsch f. rat. Med. Henle ü. Pfeiffer 3 Reihe. T. XX., p. 264.

16. *Roussin,* Annales d'Hygiène et de Med. Légale. XXIII., 1865. (Negative.)

17. *Blandlot, A.* Annales d'Hygiène, etc. XXIX., 1868. (Negative.)

18. *Neuman, A.* Die Erkennung des Blutes bei Gerichtlichen Untersuchungen, Leipzig, 1869. Also Russian Government publication, 1870. (Positive.)

19. *Richardson, J. G.*, on the detection of blood corpuscles in blood stains. Amer. Jour. Med. Sc., Vol. LVIII., 1869, p. 50. (Positive.)

20. *Cornil, et al.* (Mayet, Mialhe and Lefort), Instruction pour servir à déterminer les éléments constituants du sang dans les taches, Societé Med. Légale de

France. Annales d'Hygiène, 1873. Also Bull, Paris, 1873–4, III., p. 53–65. (Positive.)

21. *Rabuteau*, Revue de Sciences Médicales, 1874. (Negative.)

23. *Richardson, J. G.*, on the value of high powers in the diagnosis of blood stains. Am. Jour. Med. Sc., 1874, LXVIII., p. 102. Also, Philadelphia Med. Times, 1874, p. 663, 826. Also, London Med. Record, 1874, II., 560. (Positive.)

24. ———— Note on the Diagnosis of Blood Stains. Monthly Microsc. Journal, London, 1875, XIII., 213. (Positive.)

25. ———— On the Miscroscopic Means of Distinguishing the Stains of Undiluted from those of Diluted Blood. Med. and Surg. Reporter, Phila., 1869, XX., 21. Russian translation, pamphlet, St. Petersburg. 1876. (Positive.)

26. *Gulliver, G.* Observation on the Sizes and Shapes of Red blood Corpuscles of the Blood of Vertebrates, etc. Proceeding Zoölogical Soc. of London, June, 1875, p. 474. (See text and diagram.) (Positive.)

27. *Malinin, J. I.*, on Distinguishing the Blood Corpuscles of Man from those of Animals in Dried Blood Stains. Transaction of the Kaukasian Med. Soc. (Russian.) Tiflis, 1873–74, X., 298. (Positive.)

28. ———— Ibid, p. 335. (Positive.)

29. ———— Alconolic Solution of Caustic Potash as a Reagent for Examination of Blood Stains. Ibid. p. 338. (Positive.)

30. *Malinin*, Ueber die Erkennung des menschlichen Blutes in trockenen Flecken, etc. Virchow's Archiv., T. LXV., 1875, p. 528. (Positive.)

31. *Seiler. C.* Photographic Enlargements of Blood Corpuscles. Phila. Med. Times, 1875.

32. ———— High Powers in Micro-photography, Phila. Med. Times, 1875. (Positive.)

33. *Huber, A.*, Beitrag zur gerichtl, microscopischen Diagnose der Blutflecken. Würzburg, 1876.

34. *Woodward, J. J.* On the Similarity of the Red Blood Corpuscles of Man and those of other Mammals, especially the Dog, Amer. Journal Med. Sc., 1875, p. 151, and monthly Microscop. Journal, 1875, p. 65. (Negative.)

35.———— Application of Photography to Micrometry, with special reference to the Micrometry of Blood of Criminal Cases. Transaction of the American Medical Association, 1876, p. 302. See also Discussion, p. 296. (Negative.)

36. *Hemphile, W. D.* Investigation of Blood Stains in Med. Legal Investigations. Dublin Jour. Med. Sc., 1875, T., p. 330.

37. *Johnston, Christopher.* The Microscopy of Blood. Transactions of the International Medical Congress of Philadelphia, 1876; Section of Biology, p. 467. See also Discussion on above paper by Richardson and others. (Positive.)

38. *Canvet.* Annales de Hygiène et Sciences Légale, 1877, (Positive.)

39. *Richardson, J. G.* On the Identity of the Red Blood Corpuscles in Different Races of Mankind. Amer. Jour. Medical Sciences, January, 1877. (Positive.)

40. *Schmid, Hans.* Ueber die Möglichkeit der Unterscheidung des Menschlichen und thierischen Blutes in trockenen Flecken, in Gerichtlicher Beziehung, Diss. Erlangan, 1878. (Positive.)

41. *Morache.* Annales d'Hygiène, etc., 1880. p. 322. (Negative.)

42. *Struve, H.* Die Diagnostik von Blutflecken durch Messung von Blutkörperchen. Virchow's Archiv., 1881, T. 83, p. 146; Ibid, 1880, T. 79, p. 524. (Negative.)

43. *Hoffman.* Nouveaux Eléments de Médicine Légale. Paris, 1881. (Negative.)

44. *Vilbert.* Archives de Physiology, 1882. (Positive.)

45. ———— Article "Blood" in his "Nouveau Dictionair." (Positive.)

46. *Wormley, Th.* Detection and Microscopic Discrimination of Blood, Appendix to his work on Micro-Chemistry of Poisons, 2d ed., Phila. 1885. (Positive.)

47. *Masson, M.* L'Origin de Sang. Annales d'Hygiene et de Medicine Lé ale; vide also reprint. Paris, 1885. (Positive.)

48. *Lacour, E.* Considérations generales sur la recherche du sang, dans les cas d'expertises Médico-Légales. Archives de Med. and Pharmacie Militaire. Paris, 1887, X., p. 358. (Positive.)

49. *Bizzozzero.* Article on Blood in his "Klinische Microscopie," 1887.

50. *Reese, J. J.* Medical Jurisprudence and Toxicology. Also American edition of Talayor's Medical Jurisprudence, by Reese. (Positive.)

51. *Hayem.* Recherches sur l'Anatomie norm. et path. du Sang. Paris, 1878. Comptes Rendus de l'Acad. de Sc., 1882, 18 Juli.

52. *Bizzozero.* Virchow's Archiv., 1882, Bd. 90, p. [illegible]61.

53. *Osler, Wm.* On certain Problems in the Physiology of the Blood Corpuscles. The Medical News, April 3, October 17, 1886.

54. *Welch.* The Structure of White Thrombi. Transactions of the Pathological Society of Philadelphia, Vol. XIII., 1887.

55. *Sternberg, Geo. M.* Photo-micrographs and how to make them. Boston, 1884.

## Figures and Tables

**Fig. 1. f1:**
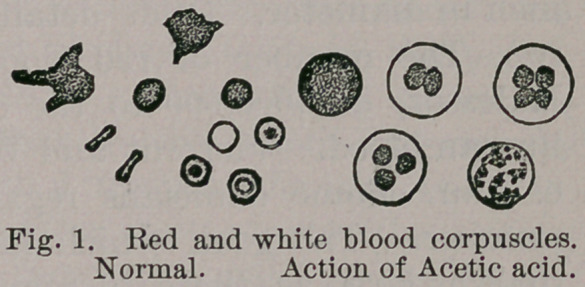


**Figure 2. f2:**
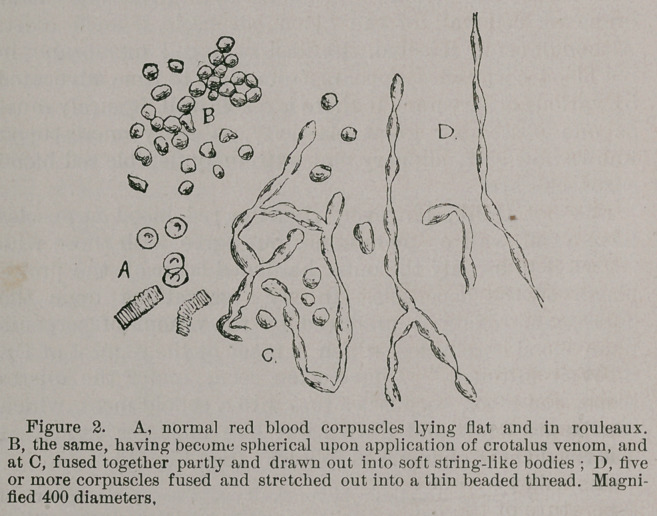


**Figure 3. f3:**
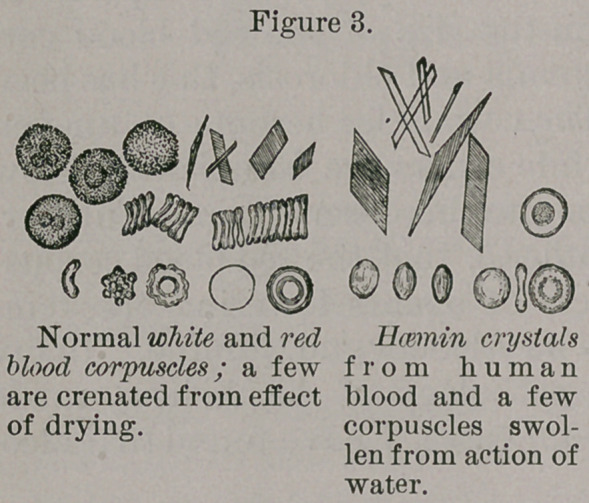


**PLATE I. f4:**
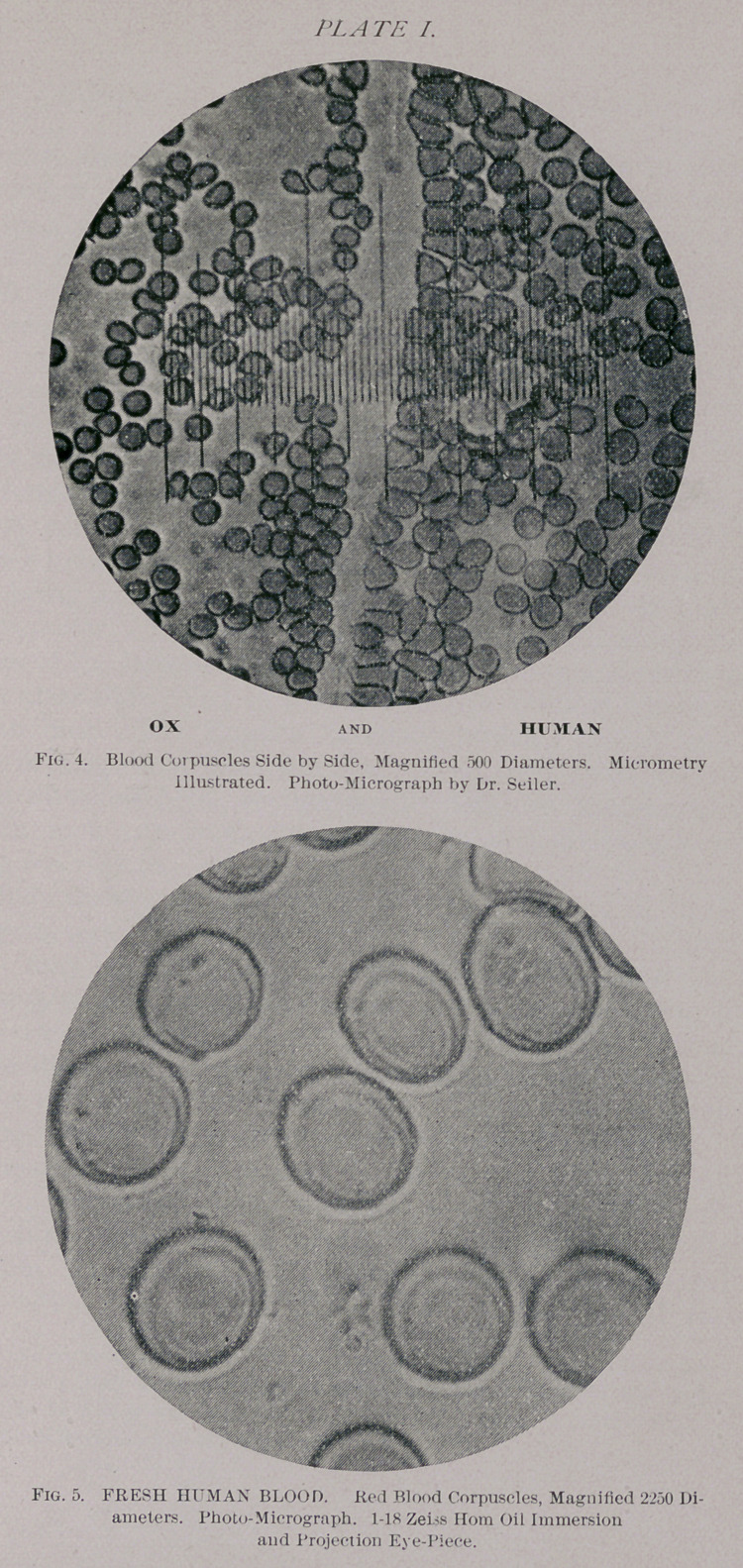


**PLATE II. f5:**
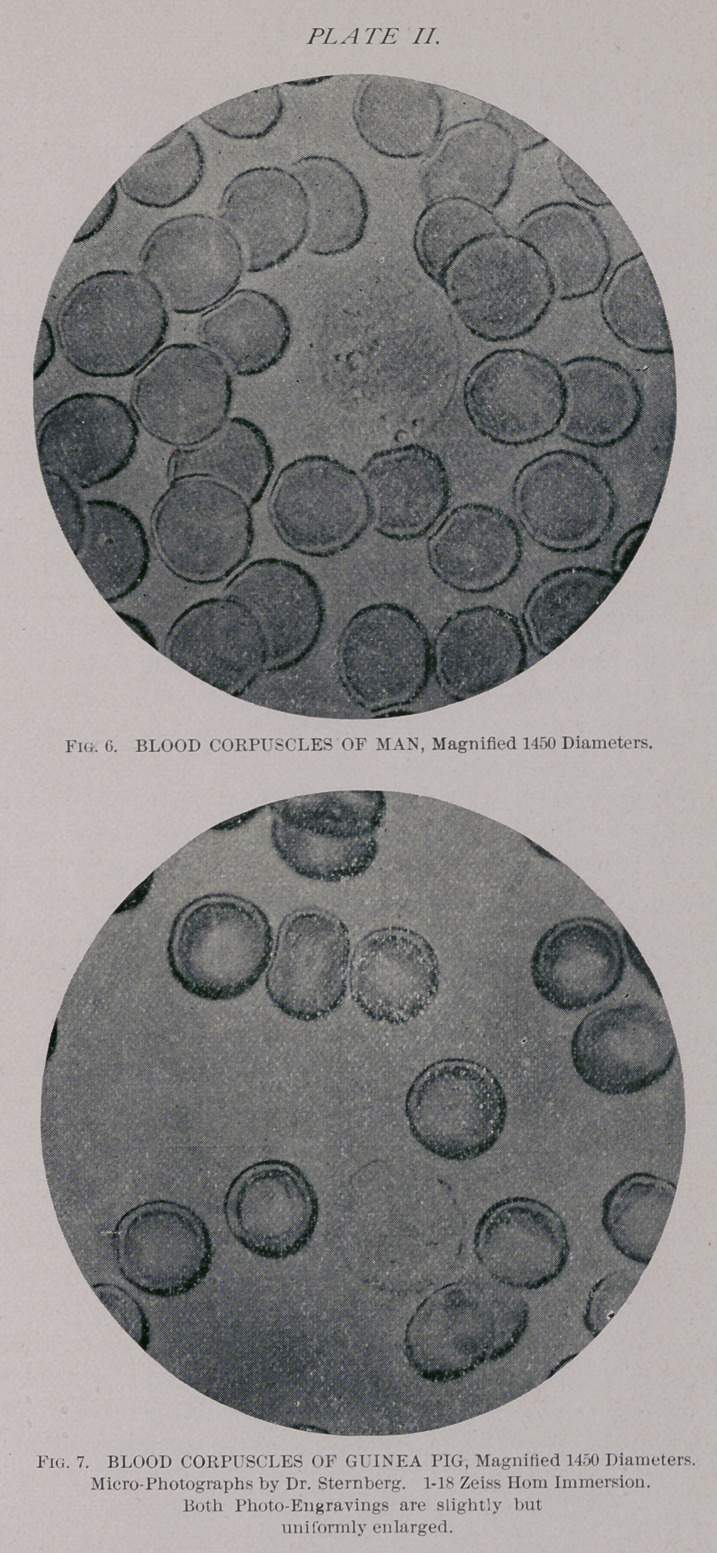


**PLATE III. f6:**
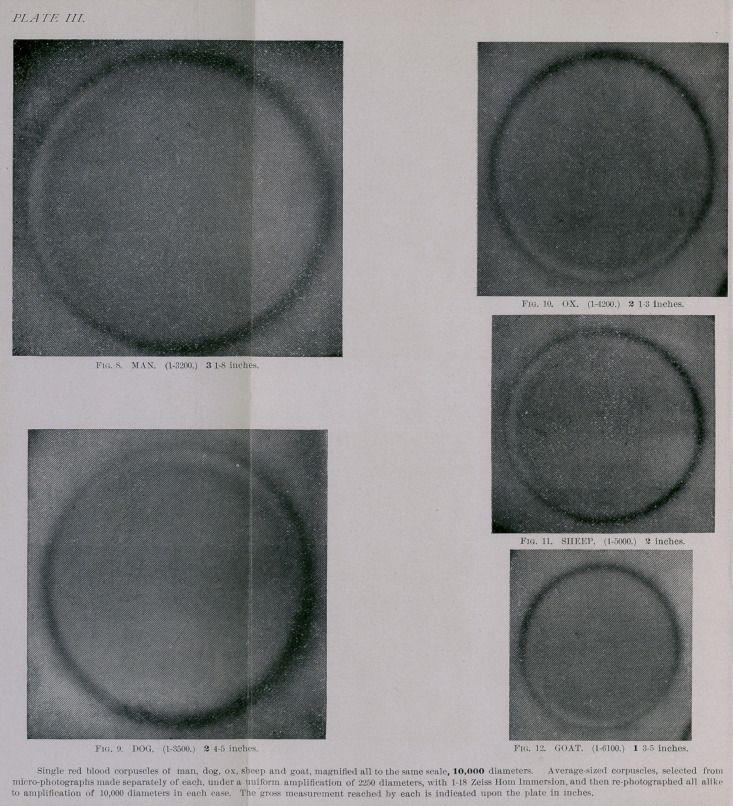


**PLATE IV. f7:**
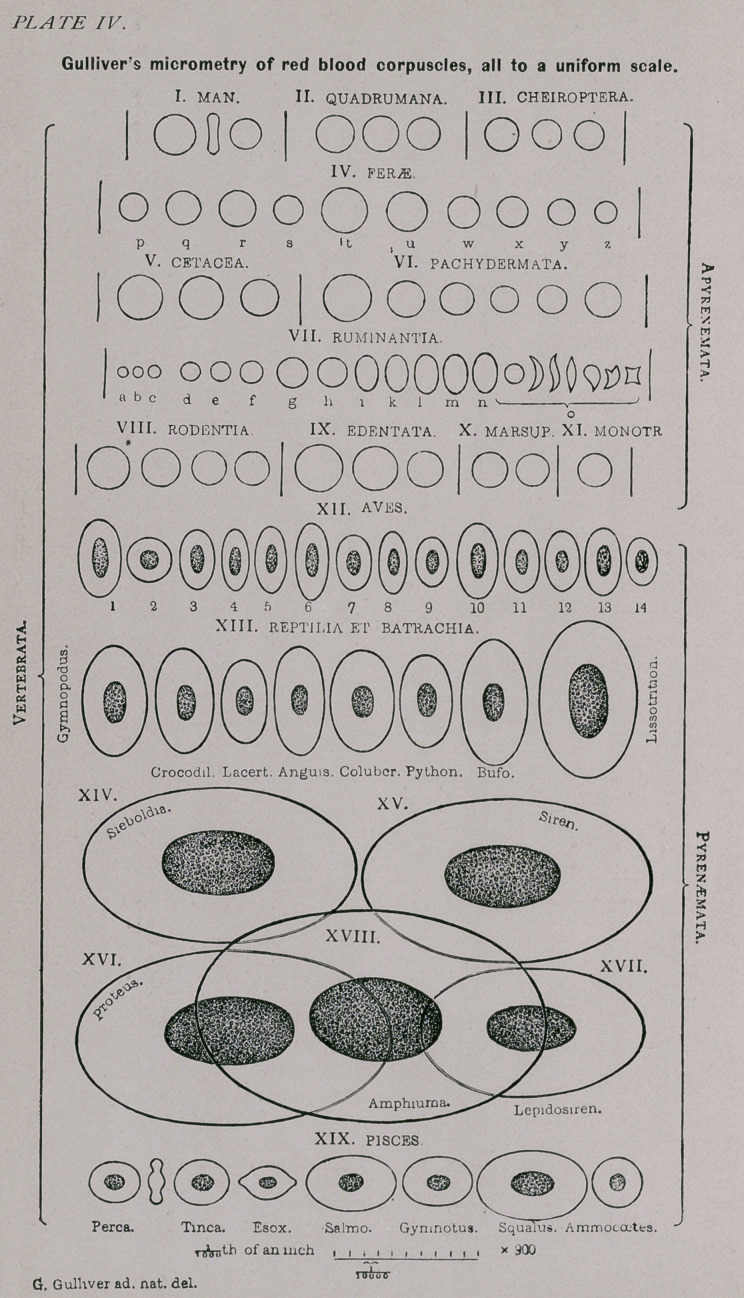


**PLATE V. f8:**
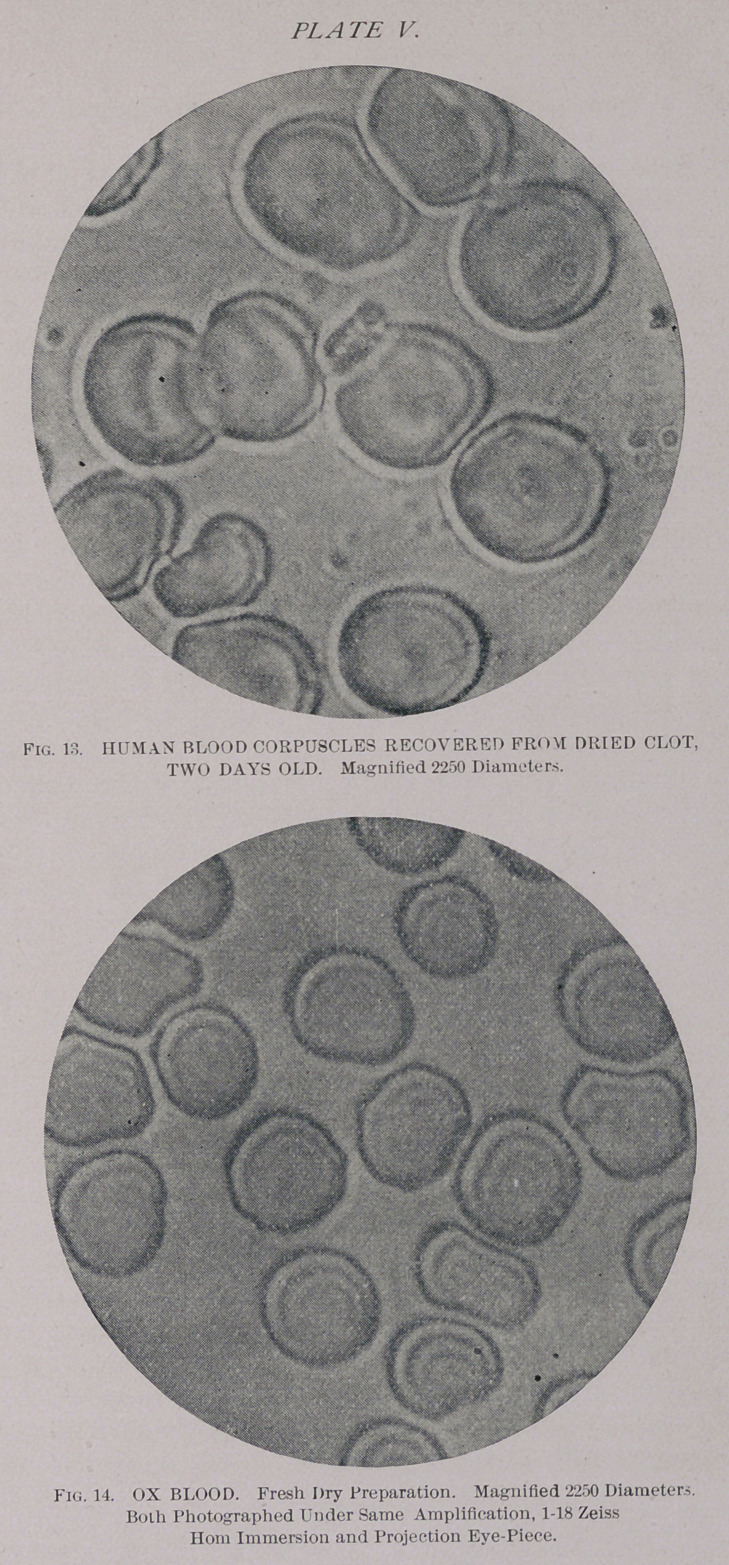


**PLATE VI. f9:**
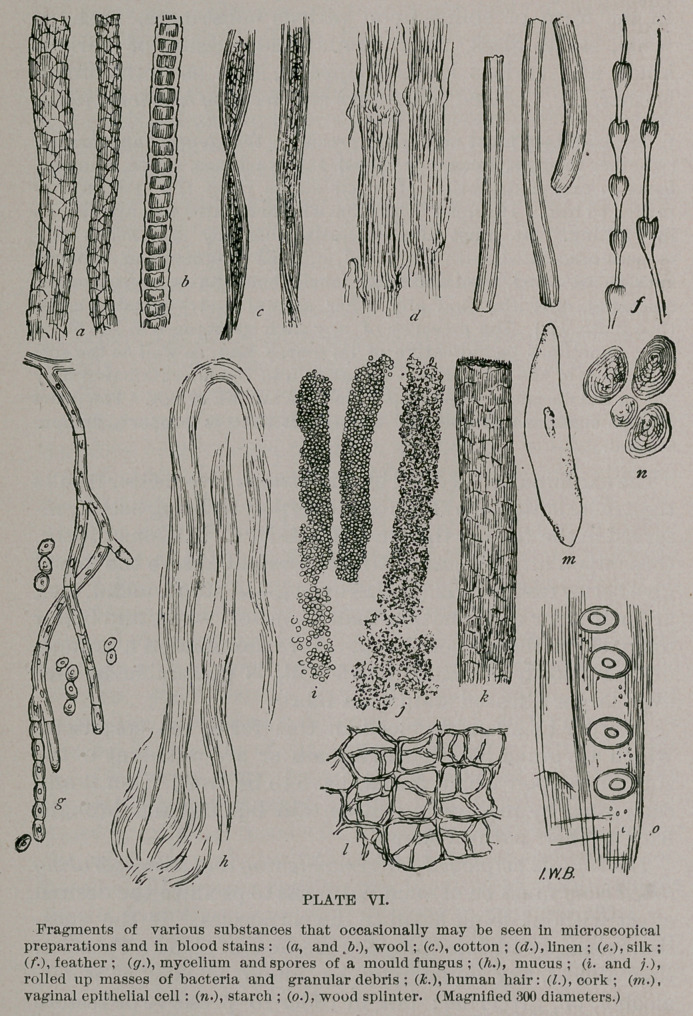


**Fig. 15. f10:**